# Supplementary material for: Redox-innocent scandium(III) as the sole catalyst in visible light photooxidations
Source: Nat Commun. 2025 Aug 22;16:7851. doi: 10.1038/s41467-025-63233-4 (PMC12373787; doi:10.1038/s41467-025-63233-4)
Supplement: Supplementary file 1 — Supplementary Information [file 41467_2025_63233_MOESM1_ESM.pdf]

Supplementary Information

Redox-innocent scandium(III) as the sole catalyst in visible light photooxidations

Amal Hassan Tolba,* Ahmed M. El-Zohry,* Jafar Iqbal Khan, Eva Svobodová, Josef Chudoba, Jiří Klíma, Karol Lušpai, Martin Pižl, Jiří Šturala, Radek Cibulka*

*Corresponding authors: ahmed.elzohry@kfupm.edu.sa; cibulkar@vscht.cz

S1 General procedures ………………………………………………………… 2

S2 Exploring and optimisation of $Sc(OTf)_3$-based aerobic photocatalytic oxidation of benzylic substrates ……………………………………………………………………… 4

S3 Photocatalytic aerobic oxidations – substrate scope investigation……………… 7

S4 Optimisation of photocatalytic oxidative cyanation of arenes using $Sc(OTf)_3$ under aerobic conditions……………………………………………………………………… 12

S5 Preparation of 1-methoxy-2-(trifluoromethyl)benzene (**18h**)……………………… 14

S6 Photocatalytic oxidative cyanation – substrate scope investigation………………… 16

S7 Radical trapping and fluorescence quenching experiments………………………… 18

S8 Experiment with isotopically labelled oxygen……………………………………… 24

S9 Cyclic voltammetry measurements ……………………………………………… 27

S10 EPR measurements…………………………………………………………… 28

S11 Determination of hydrogen peroxide in the reaction mixture……………………… 29

S12 Quantum chemical calculations – UV-Vis spectra and excited states…………… 30

S13 NMR spectra of photooxidation and cyanation products………………………… 33

S14 Experimental setup of photocatalytic experiments………………………………… 43

References………………………………………………………………………… 45

S1. General procedures

Materials and Instrumentation: chemicals were purchased from Sigma-Aldrich and Fluorochem. The solvents were purified and dried using standard procedures. Commercially obtained reagents were used as received without further purification unless otherwise stated. Thin layer chromatography (TLC) analyses were carried out on DC Alufolien Kieselgel 60 F254 (Merck). The compounds were visualised with UV light (254 and 366 nm). **Nuclear magnetic resonance** (NMR) spectra were recorded on a Agilent 400-MR DDR2 (399.94 MHz for ^{1}H, 100.58 MHz for ^{13}C), or JNM-ECZL400S spectrometer (JEOL Ltd., 399.94 MHz for ^{1}H, 100.58 MHz for ^{13}C) at 298 K. Data for ^{1}H NMR are reported as follows: chemical shift (δ ppm), multiplicity (s = singlet, d = doublet, t = triplet, q = quartet, m = multiplet, dd = doublet of doublets, dt = doublet of triplets, br = broad etc.), coupling constant (Hz), and integration. ^{19}F NMR chemical shifts were measured relative to CCl_3F. All NMR spectra were processed and assigned using MestreNova. **High resolution mass spectra** were obtained on LTQ Orbitrap Velos (Thermo Scientific), equipped with an ion trap and orbitrap analyser. Electrospray (ESI) and Atmospheric Chemical Ionisation (APCI) high resolution mass spectra were obtained on LTQ Orbitrap Velos (Thermo Scientific), equipped with a ion trap and orbitrap analyser. GC/MS analyses were performed on Trace Ultra gas chromatograph connected with a single quadrupole DSQ II mass spectrometer (Thermo Scientific) equipped with an electron ionisation (EI+ 70eV) ion source. DB-5 30 m × 0.25 mm, film 0.25 um GC column (Agilent) was used. The **melting points** were measured on a Boetilus melting point apparatus and are uncorrected. **UV-Vis absorption spectra** were recorded on the Agilent Cary 8454 spectrometer using quartz cells of 1 cm. **Fluorescence emission spectra** were measured using Varian Eclipse spectrometer. The fluorescence quenching of $Sc(OTf)_3$ in acetonitrile by an electron donor (substrate) was measured in MeCN at 25 °C. Stern-Volmer plots ($I_o/I=1+K_S[Q]$) were constructed, and the constant K_S was evaluated as the slope of the dependence by using OriginPro 2021 64-bit software. **EPR spectra** were recorded by X-band EPR spectrometer EMX micro (Bruker, Germany) with 100 kHz field modulation using Bruker EPR high sensitivity resonator ER 4119 HS. The g-values of the radical species were determined using BDPA external standard. The experimental EPR spectra were processed by the Bruker software Xenon. A flat (0.5 mm) quartz cuvette was used. The sample was irradiated in situ, i.e. in the resonator of the EPR spectrometer by 400 nm LEDs. For experiments under inert atmosphere, the oxygen was removed by bubbling with argon pre-saturated by MeCN for about 10 minutes. **Time-resolved photoluminescence**: The time-resolved emission

experiments were conducted using the time-correlated single-photon counting technique (TCSPC). The TCSPC consists of a pulsed laser diode source at 377 nm with a pulse width of ca. 70 ps at a repetition rate of 100 MHz provided from Horiba (Delta Diode) coupled with a controller box (DD-C1). The emission is collected at 90° using a Nano log instrument (Horiba) and synchronised with the diode box. The fitting lifetimes were extracted using the instrument software with exponential equations coupled with the instrument response function. Femtosecond laser setup: The femtosecond laser setup for transient absorption is described as follows: seeding short laser pulses (MaiTai-Spectra Physics) at a wavelength of 800 nm with a bandwidth of 15 nm and a repetition rate of 85 MHz provides an output power of 0.78 W. The seed pulse is stretched several times, reaching ca. 200 ps pulse width before entering the amplifier cavity (Spitfire, Spectra-Physics). After several rounds inside the cavity (ca. 16 rounds, each takes ca. 7.2 ns), the amplified pulse is ejected using pockel cells and compressed again to ca. 100 fs pulse width with an average energy per pulse of 3.5 μJ/pulse (2 kHz). The amplified pulse is then split into two paths using a beam splitter and directed into two TOPASs (light conversion) to generate both the pump and the probe pulses. The pump pulse is passed through a chopper device and then focused on the moving quartz cell containing the sample. The pump pulse has a radius of 210 μm and an energy of 250 nJ at 400 nm. The probe pulse is selected to be at 780 nm to be focused into a rotating CaF_2 crystal with 3 mm thickness, which generates white light (WL) ranging from 350 to 720 nm. The WL is split into two paths: one for the sample and the other one for reference. The first WL part with a high intensity of ca. 150 nJ and a beam radius of ca. 120 μm is spatially and temporarily overlapped on the moving sample with the pump beam at the selected wavelength. The transmitted pump pulse is blocked, while both the transmitted WL and the reference WL are directed into two CCDs (FL3000-Stresing) coupled with monochromators (Acton Series-Princeton Instruments). Using a homemade LabVIEW interface, both WL signals are corrected to the background and fluctuations. Then, a homemade Matlab script is implemented to do the proper analysis. **Photochemical Setup**: Reactions were performed in vessels from borosilicate glass using commercial LED(s) as a light source: LED Engine, 1.35 W@700 mA, 400 nm (dominant wavelengths 385–410 nm) for analytical experiments and 8 × 1.35 W@700 mA, 400 nm for preparative experiments; see S15 for arrangement.

S2 Exploring and optimisation of $Sc(OTf)_3$-based aerobic photocatalytic oxidation of benzylic substrates

Table S1. Screening of Lewis acids for photocatalytic aerobic oxidation of various benzylic substrates [a]

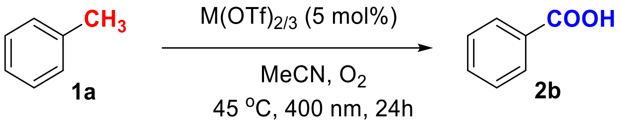

Entry	Lewis acid $M(OTf)_n$	Yield[b] [%]
1	$Sc(OTf)_3$	80
2	$Mg(OTf)_2$	n.d.[c]
3	$Zn(OTf)_2$	n.d. [c]
4	$La(OTf)_3$	n.d. [c]
5	$Ba(OTf)_2$	n.d. [c]

[a] Standard reaction conditions: Substrate (0.140 mmol), $M(OTf)_{2/3}$ (5 mol%), MeCN (0.25 mL), 45 °C, O_2 (balloon), blue LED (400 nm), and 24 h. [b] Yields were determined from ^1H NMR spectra. [c] Product was not detected.

Table S2. Screening of catalyst loading for photocatalytic aerobic oxidation of various benzylic substrates [a]

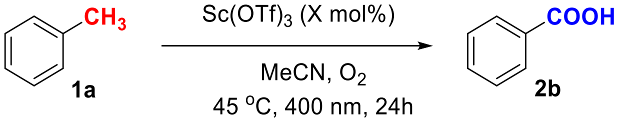

Entry	$Sc(OTf)_3$ loading [mol%]	Yield[b] [%]
1	2.5	46
2	5	74
3	10	83

[a] Standard reaction conditions: Substrate (0.140 mmol), MeCN (0.25 mL), 45 °C, O_2 (balloon), blue LED (400 nm), and 24 h. [b] Yields were determined from ^1H NMR spectra.

Table S3. TOF for Sc(OTf)3 as a photocatalyst in the direct aerobic oxidation of toluene[a]

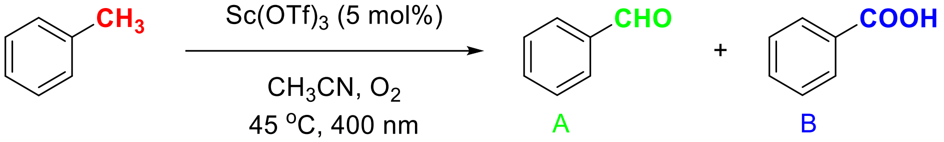

Entry	Time [h]	A [%]	B [%]	Yield[b] [%]	No.of mmoles of PhCOOH	TON[d]	TOF[e] [h^{-1}]
1	4	3	9	12	0.0169	2.4	0.600
2	8	3	30	33	0.0465	6.6	0.825
3	12	3	49	52	0.0733	10.5	0.875
4	16	3	60	63	0.0888	12.7	0.794
5	20	n.d.[c]	86	86	0.1213	17.3	0.865

[a] Standard reaction conditions: (0.140 mmol), Sc(OTf)3 (5 mol%), MeCN (0.25 mL), 45 °C, O_2 (balloon), blue LED (400nm), and 24 h. [b] Yields were determined from ^{1}H NMR spectra.[c] Product was not detected. [d] Turnover number = No. of moles of the product / No. of moles of Sc(OTf)3 loading. [e] Turrover frequency: TON of Sc(OTf)3 during a reaction over a specified time period.

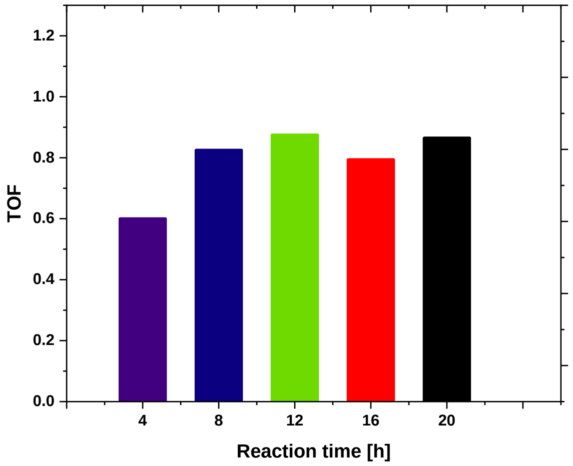

Figure S1. Turnover frequency (TOF) for Sc(OTf)3 photocatalyst in the direct aerobic oxidation of toluene.

Table S4. Screening of acids to allow oxidations of 4-trifluoromethyltoluene (**1c**)[a]

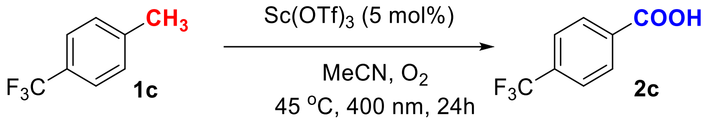

Entry	Conditions alternation	Yield[b] [%]
1	-	traces
2	+CF_3COOH (0.2 equiv.), EtCN solvent, 65 °C	25
3	+CF_3COOH (0.2 equiv.), EtCN solvent, 65 °C, 48 h	33
4	+CF_3COOH (1 equiv.), EtCN solvent, 65 °C	13
5	+CF_3COOH (0.2 equiv.), EtCN solvent, 65 °C, no $Sc(OTf)_3$	0
6	+CF_3COOH (1 equiv.), EtCN solvent, 65 °C, no $Sc(OTf)_3$	1
7	+CH_3COOH (1 equiv.), EtCN solvent, 65 °C	3
8	+TfOH (1 equiv.), EtCN solvent, 65 °C	0
9	+CH_3COOH (1 equiv.), EtCN solvent, 65 °C, no $Sc(OTf)_3$	0

[a] Standard reaction conditions: Substrate (0.140 mmol), MeCN (0.25 mL), 45 °C, O_2 (balloon), blue LED (400 nm), and 24 h. [b] Yields were determined from ^1H and ^{19}F NMR spectra.

S3 Photocatalytic aerobic oxidations – substrate scope investigation

General procedure A: preliminary experiments

A vial was charged with a mixture of the substrate (140 µmol) and $Sc(OTf)_3$ (5 mol%, 7 µmol) in MeCN (250 µL). The reaction mixture was bubbled with oxygen (2 min) and then stirred at 45 °C under irradiation with 400 nm LEDs. When the reaction was completed, it was diluted with DMSO-d_6 and the yield was determined by ^1H NMR.

General procedure B: preparative experiments

A mixture of the substrate (1 mmol) and $Sc(OTf)_3$ (5 mol%) in MeCN (5 mL) was bubbled with oxygen (2 min). It was then stirred at ambient temperature in a 50 mL borosilicate glass Schlenk tube under irradiation with 400 nm LEDs under aerobic conditions (balloon). The reaction progress was monitored by ^1H NMR analysis of the reaction mixture. When the reaction was completed, the solvent was evaporated, and the crude product was purified either by column chromatography on silica gel or by extraction (**see below**).

General procedure of purification by extraction

The product was extracted between 1M NaOH (20 mL) and Et_2O (20 mL). The organic phase was extracted by 1M NaOH (2 × 5 mL). Combined water phases were washed by Et_2O (20 mL), acidified (conc. HCl), and finally extracted by Et_2O (3 × 20 mL). Combined organic phases from the last extraction (3 × 20 mL Et_2O) were dried over $MgSO_4$. Then drying agent was removed by filtration and the filtrate was evaporated.

Oxidation of toluene

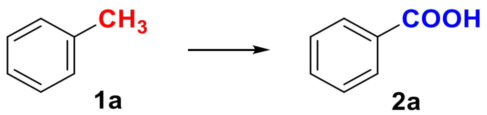

1a **2a**

Toluene (**1a**; 1 mmol, 92 mg) was oxidised using $Sc(OTf)_3$ (0.05 mmol, 25 mg) by the general procedure **B**.

Reaction time: 48 h.

Purification: extraction, yielding **2a** as a white solid.

Isolated yield: 83% (101 mg).

^{1}H NMR (400 MHz, DMSO-d$_6$) δ 12.97 (s, 1H), 7.95 (d, *J* = 7.7 Hz, 2H), 7.61 (t, *J* = 7.3 Hz, 1H), 7.49 (t, *J* = 7.5 Hz, 2H). **^{13}C NMR** (101 MHz, DMSO-d$_6$) δ 168.2, 133.4, 131.3, 129.8, 129.1 ppm. **HRMS (APCI$^-$)**: For $C_7H_5O_2^-$ [M-H]$^-$ calculated 121.02950, found 121.02897.
m.p. 118–120 °C (ref.[1] 118–120.4 °C).

Oxidation of trans-stilbene

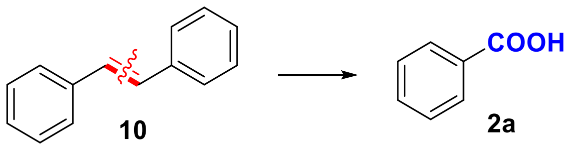

trans-Stilbene (**10**; 1 mmol, 180 mg) was oxidised using Sc(OTf)$_3$ (0.05 mmol, 25 mg) by general procedure **B**.
Reaction time: 72 h.
Purification: extraction, yielding **2a** as a white solid.
Isolated yield: 57% (139 mg).

^{1}H NMR spectrum of **2a** corresponded to that above.

Oxidation of p-xylene

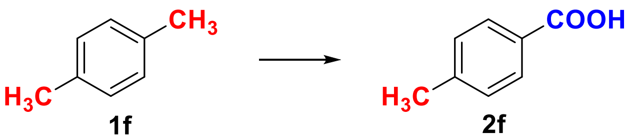

p-Xylene (**1f**; 1 mmol, 106 mg) was oxidised using Sc(OTf)$_3$ (5 mol%, 25 mg) by general procedure **B**. The product was isolated by extraction.
Reaction time: 24 h
Purification: extraction, yielding **2f** as an off-white solid.
Isolated yield: 99% (135 mg)
^{1}H NMR (400 MHz, DMSO-d$_6$) δ 12.82 (s, 1H), 7.84 (d, *J* = 8.0 Hz, 2H), 7.30 (d, *J* = 8.4 Hz, 2H), 2.37 (s, 3H). **^{13}C NMR** (101 MHz, DMSO-d$_6$) δ 167.8, 143.6, 129.9, 129.7, 128.5, 21.7 ppm. **HRMS (APCI$^-$)**: For $C_8H_7O_2^-$ [M-H]$^-$ calculated 135.04515, found 135.04463.
m.p. 177–178 °C (ref.[2] 177–180 °C)

Oxidation of 9H-fluorene

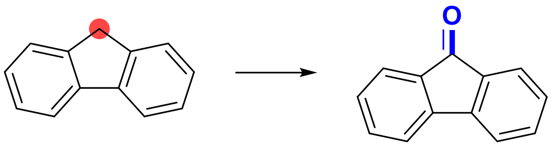

8 9

9*H*-Fluorene (**8**; 1 mmol, 166 mg) was oxidised using Sc(OTf)$_3$ (5 mol%, 25 mg) by general procedure **B**.

Reaction time: 24 h

Purification: column chromatography (hexane:ethyl acetate, 20:1), yielding **9** as a yellow solid.

Isolated yield: 74% (133 mg)

^{1}H NMR (400 MHz, CDCl$_3$) δ 7.63 (d, *J* = 7.3 Hz, 2H), 7.46 (q, *J* = 7.4, 7.3 Hz, 4H), 7.26 (t, J = 7.0 Hz, 2H). **^{13}C NMR** (101 MHz, CDCl$_3$) δ 194.2, 144.5, 134.8, 134.2, 129.1, 124.3, 120.4 ppm. **HRMS (APCI$^+$)**: For $C_{13}H_9O^+$ [M+H]$^+$ calculated 181.06479, found 181.06488. **m.p.** 77–80 °C (ref.[3] 79–81 °C).

Oxidation of benzyl methyl ether

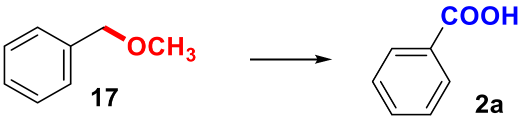

17 2a

Benzyl methyl ether (**17**; 1 mmol, 122 mg) was oxidised using Sc(OTf)$_3$ (5 mol%, 25 mg) by general procedure **B**.

Reaction time: 24 h

Purification: extraction, yielding **2a** as an off-white solid.

Isolated yield: 51% (62 mg)

^{1}H NMR spectrum of **2a** corresponded to that above.

Oxidation of 1-(4-chlorophenyl)ethanol

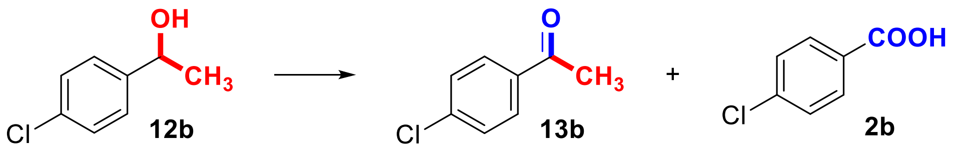

1-(4-Chlorophenyl)ethanol_(**12b**; 1 mmol, 156 mg) was oxidised using $Sc(OTf)_3$ (5 mol%, 25 mg) by general procedure **B**.

Reaction time: 24 h

Purification: column chromatography (hexane:ethyl acetate, 20:1), yielding a mixture of **13b** as a colourless liquid and **2b** as a white solid.

Isolated yield: 59% (91 mg) of **13a** and 16% (25 mg) of **2b**.

*4-Chloroacetophenone (**13a**)*: **^{1}H NMR** (400 MHz, $CDCl_3$) δ 7.85 (d, J = 8.5 Hz, 2H), 7.38 (d, J = 8.5, 2H), 2.55 (s, 3H). **^{13}C NMR** (101 MHz, $CDCl_3$) δ 196.61, 139.58, 135.45, 129.79, 128.92, 26.41 ppm. **HRMS (APCI$^+$)**: For $C_8H_8ClO^+$ $[M+H]^+$ calculated 155.02585, found 155.02591.

*4-Chlorobenzoic acid (**2b**)*: **^{1}H NMR** (400 MHz, DMSO-d_6) δ 13.19 (s, 1H), 7.94 (d, J = 8.5 Hz, 2H), 7.57 (d, J = 8.5 Hz, 2H). **^{13}C NMR** (101 MHz, DMSO-d_6) δ 166.9, 138.3, 131.7, 130.1, 129.3 ppm. **HRMS (APCI$^-$)**: For $C_7H_4O_2^-$ $[M-H]^-$ calculated 154.99053, found 154.99016.

Oxidation of 1-chloro-4-ethylbenzene

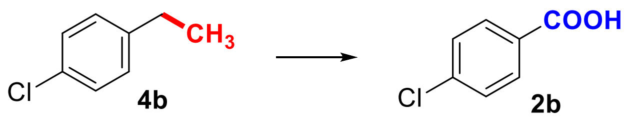

1-Chloro-4-ethylbenzene (**4b**; 1 mmol, 156 mg) was oxidised using $Sc(OTf)_3$ (5 mol%, 25 mg) by general procedure **B**.

Reaction time: 30 h

Purification: extraction; yielding **2b** as a white solid.

Isolated yield: 59% (105 mg)

m.p. 234–236 °C (ref.[2] 238–241 °C)

^{1}H NMR spectrum of **2b** corresponded to that above.

Oxidation of methyl 4-methylbenzoate

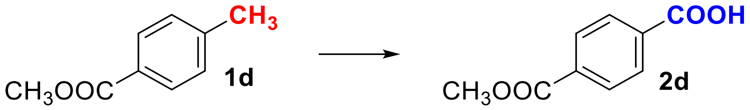

Methyl 4-methylbenzoate (**1d**; 1 mmol, 150 mg) was oxidised using $Sc(OTf)_3$ (5 mol%, 25 mg) by general procedure **B**.

Reaction time: 24 h

Purification: extraction, yielding **2d** as an off-white solid.

Isolated yield: 61% (110 mg).

^{1}H NMR (400 MHz, DMSO-d_6) δ 13.19 (s, 1H), 8.11 (s, 4H), 3.88 (s, 3H). **^{13}C NMR** (101 MHz, DMSO-d_6) δ 167.1, 166.1, 135.3, 133.7, 130.1, 129.9, 53.0 ppm. **HRMS (APCI$^-$)**: For $C_9H_7O_4^-$ $[M\text{-}H]^-$ calculated 179.03498, found 179.03505.

m.p. 219–221 °C (ref.[4] 219–221 °C).

S4 Optimisation of photocatalytic oxidative cyanation of arenes using $Sc(OTf)_3$ under aerobic conditions

Table S5. Effect of the concentration of TMSCN on the yield and reaction time of cyanation of biphenyl (**18e**) mediated by $Sc(OTf)_3$ as a photocatalyst

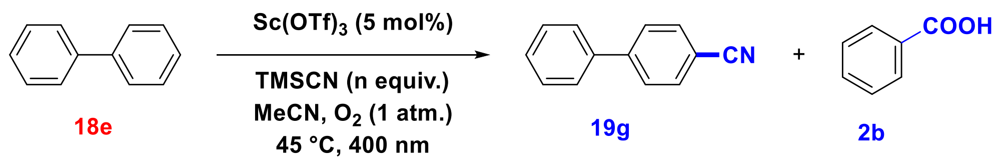

Entry	TMSCN (no. of equiv.)	Reaction time [h]	Yield [%]	
			**19g**	**2b**
1	0	24	n.d.	80
2	4	10	41	traces
3	4	24	47	traces
4	4	48	43	36
5	12	6	quant.	n.d.

Table S6. Screening of Lewis acids for photocatalytic direct oxidative cyanation of arenes [a]

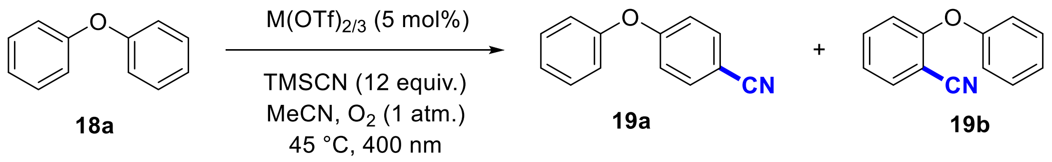

Entry	Lewis acid $M(OTf)_{2/3}$	Yield[b] [%] $M(OTf)_{2/3}$
1	$Sc(OTf)_3$	74
2	$Mg(OTf)_2$	n.d.[c]
3	$Zn(OTf)_2$	n.d. [c]
4	$La(OTf)_3$	n.d. [c]
5	$Ba(OTf)_2$	n.d. [c]

[a] Standard reaction conditions: Substrate (0.140 mmol), $M(OTf)_{2/3}$ (5 mol%), TMSCN (12 equiv., 1.68 mmol), MeCN (0.25 mL), 45 °C, O_2 (balloon), blue LED (400 nm), and 24 h. [b] Yields were determined from ^1H NMR spectra. [c] Product was not detected.

Table S7. Screening of catalyst loading for photocatalytic direct oxidative cyanation of arenes [a]

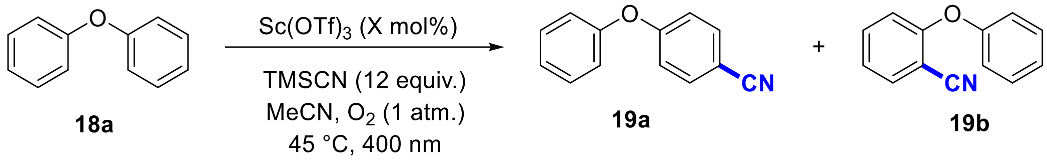

Entry	$Sc(OTf)_3$ loading [mol%]	Yield[b] [%]
1	2.5	46
2	5	74
3	10	83

[a] Standard reaction conditions: Substrate (0.140 mmol), TMSCN (12 equiv., 1.68 mmol), MeCN (0.25 mL), 45 °C, O_2 (balloon), blue LED (400 nm), and 24 h. [a] Yields were determined from ^{1}H NMR spectra. [c] Product was not detected.

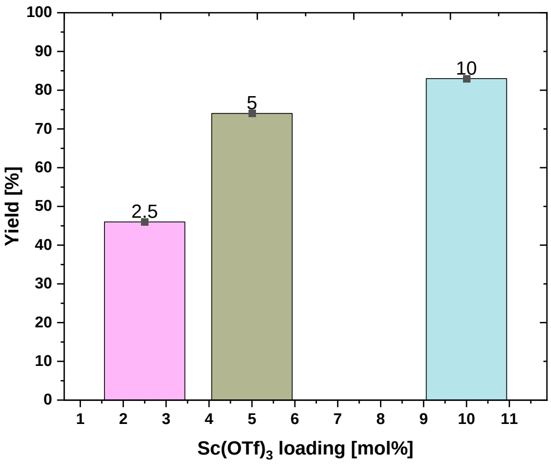

Figure S2. $Sc(OTf)_3$ loading-dependent yield for the direct photooxidative cyanation of diphenyl ether (**18a**) under aerobic conditions.

S5 Preparation of 1-methoxy-2-(trifluoromethyl)benzene (18h)

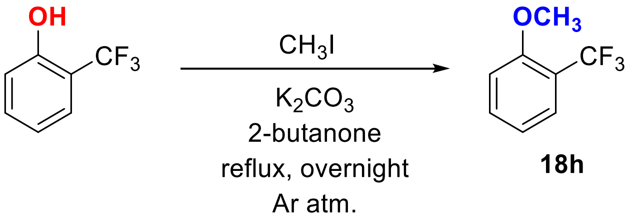

2-Hydroxybenzotrifluoride (**36**; 1000 mg, 6 mmol) was dissolved in butan-2-one (5 mL) and then was treated with K_2CO_3 (1100 mg, 8 mmol) and CH_3I (0.6 mL, 9.6 mmol). The mixture was heated at reflux overnight under an argon atmosphere until a complete reaction was indicated by thin layer chromatography (hexane: ethyl acetate, 3:1). The mixture was cooled to room temperature and filtered through diatomaceous earth. The filtered solid was washed with acetone, and the filtrate was concentrated cautiously under *vacuo*. Subsequently, the crude produce was distilled off using Kugelrohr distillation at 95–100 °C/5 torr), yielding the product **18h** as a colourless oil (729 mg, 74%).

^{1}H NMR (400 MHz, $CDCl_3$) δ 7.61 (d, J = 8.0 Hz, 1H), 7.52 (t, J = 8.0 Hz, 1H), 7.07 - 6.99 (m, 2H), 3.91 (s, 3H). **^{13}C NMR** (101 MHz, CDC13) δ 157.5 (q, $J_{C,F}$ = 1.8 Hz), 133.4, 127.0 (q, $J_{C,F}$ = 5.3 Hz), 123.9 (q, $J_{C,F}$ = 272.0 Hz), 119.9, 118.6 (q, $J_{C,F}$ = 30.7 Hz), 111.9, 55.7. **GCMS (EI+)** calculated for $C_8H_7F_3O$ $[M]^+$: 176.0449, found: 176.0447.

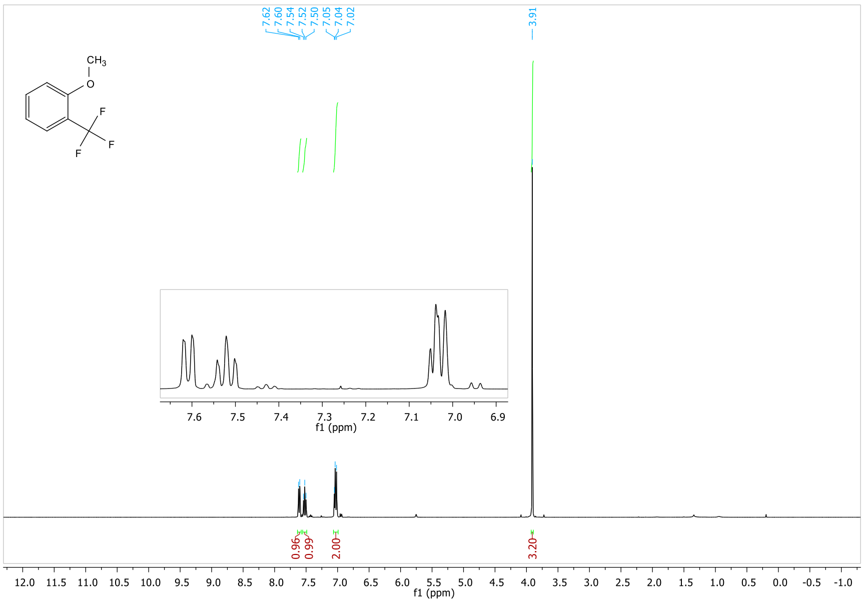

Figure S3. ^{1}H NMR (400 MHz) spectrum of methoxy-2-(trifluoromethyl)benzene (**18h**) in $CDCl_3$ at 298 K.

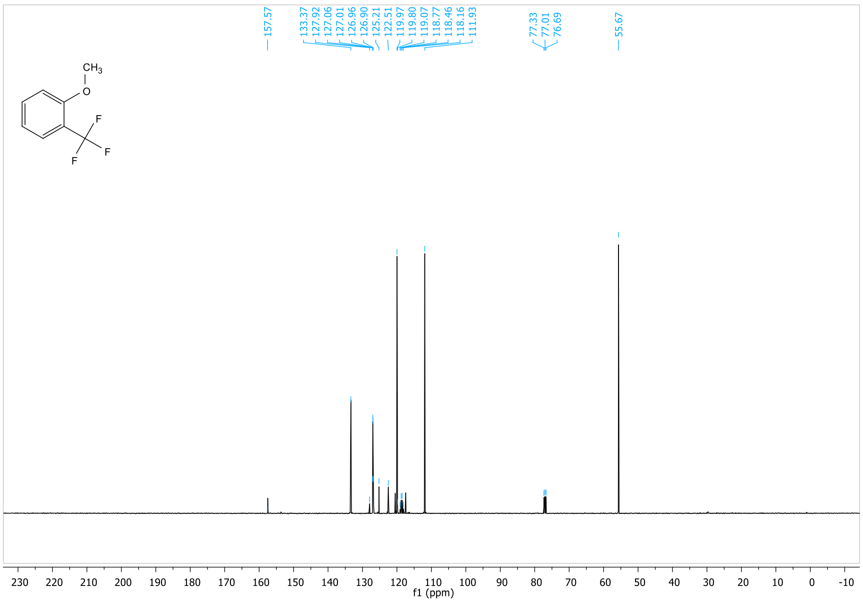

Figure S4. ^{13}C NMR (101 MHz) spectrum of methoxy-2-(trifluoromethyl)benzene (**18h**) in $CDCl_3$ at 298 K.

S6 Photocatalytic oxidative cyanation – substrate scope investigation

General procedure C: preliminary experiments

A vial was charged with a mixture of the substrate (140 µmol), $Sc(OTf)_3$ (5 mol%, 7 µmol) and TMSCN (12 equiv., 1.68 mmol) in MeCN (250 µL). The reaction mixture was bubbled with oxygen (2 min) and then stirred at 45 °C under irradiation with 400 nm LEDs. When the reaction was completed, it was diluted with DMSO-d_6 and the yield was determined by ^{1}H NMR.

General procedure D: preparative experiments

A mixture of the substrate (1 mmol), $Sc(OTf)_3$ (5 mol%) and TMSCN as a cyanating agent (12 equiv., 12 mmol, 1501 µL) in MeCN (5 mL) was bubbled with oxygen (2 min). It was then stirred at ambient temperature in a 50 mL borosilicate glass Schlenk tube under irradiation by 400 nm Luxeon LEDs under aerobic conditions (balloon). The reaction progress was monitored by TLC and ^{1}H NMR analysis of the reaction mixture. Upon the evaporation of the solvent, the crude product was purified by column chromatography on silica gel.

Cyanation of diphenyl ether (18a)

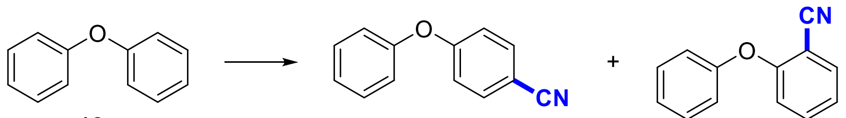

*4-Phenoxybenzonitrile (**19a**) and 2-Phenoxybenzonitrile (**19b**):* Prepared via the general procedure **D** using diphenyl ether (**18a**; 1 mmol, 170 mg), $Sc(OTf)_3$ (5 mol%, 25 mg) and TMSCN (12 eq., 1191 mg, 1501 µL) in MeCN (5 mL).

Reaction time: 36 h

Purification: column chromatography (hexane:ethyl acetate, 10:1), yielding **19a** and **19b** as pale yellow oils.

Isolated yield: 56% (**19a**:**19b**, 6:1); 48% (94 mg) of **19a**, and 8% (16 mg) of **19b**.

*4-Phenoxybenzonitrile **19a***: **^{1}H NMR** (400 MHz, $CDCl_3$) δ 7.57 (d, J = 9.0 Hz, 2H), 7.39 (d, J = 8.5 Hz, 2H), 7.22 (t, J = 7.4 Hz, 1H), 7.05 (d, J = 8.6 Hz, 2H). **^{13}C NMR** (101 MHz, $CDCl_3$) δ 161.9, 155.0, 134.4, 130.5, 125.4, 120.7, 119.1, 118.1, 106.0 ppm. **HRMS (APCI$^+$)**: For $C_{13}H_{10}NO^+$ $[M+H]^+$ 196.07569, found 196.07584.

*2-Phenoxybenzonitrile **19b***: **^{1}H NMR** (400 MHz, $CDCl_3$) δ 7.66 (d, *J* = 9.7 Hz, 1H), 7.49-7.44 (m, 1H), 7.43-7.38 (m, 2H), 7.22 (t, *J* = 8.0 Hz, 1H), 7.14 (d, *J* = 8.6 Hz, 1H), 7.09 (d, *J* = 9.7 Hz, 2H), 6.86 (d, *J* = 8.5 Hz, 1H). **^{13}C NMR** (101 MHz, $CDCl_3$) δ 159.8, 155.0, 134.2, 133.9, 130.1, 125.0, 122.8, 120.1, 116.9, 116.0, 103.7 ppm. **HRMS (APCI$^+$)**: For $C_{13}H_{10}NO^+$ $[M+H]^+$ 196.07569, found 196.07591.

Cyanation of 3-chloroanisole using $Sc(OTf)_3$ as a photocatalyst

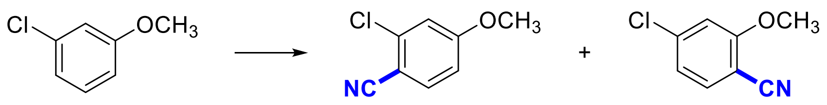

18d 19e 19f

*2-Chloro-4-methoxybenzonitrile (**19e**) and 4-chloro-2-methoxybenzonitrile (**19f**):* Prepared via the general procedure **D** using 3-chloroanisole (**18d**; 1 mmol, 142 mg), $Sc(OTf)_3$ (5 mol%, 25 mg) and TMSCN (12 eq., 1191 mg, 1501 μL) in MeCN (5 mL).

Reaction time: 32 h

Purification: column chromatography (hexane:ethyl acetate, 5:1), yielding **19f** as white crystals and a mixture of **19e** and **19f** as white solid.

Isolated yield: 75% (125 mg) as a total yield of cyanation products including **19e** and **19f** (4:1).

*2-Chloro-4-methoxy benzonitrile (**19e**)*: **^{1}H NMR** (400 MHz, $CDCl_3$) δ 7.55 (d, *J* = 8.7 Hz, 1H), 6.98 (d, *J* = 2.5 Hz, 1H), 6.85 (dd, *J* = 8.7, 2.5 Hz, 1H), 3.85 (s, 3H). **^{13}C NMR** (101 MHz, $CDCl_3$) δ 163.4, 138.2, 135.0, 116.4, 115.6, 113.5, 104.9, 56.0 ppm. **HRMS (APCI$^+$)**: For $C_8H_7ClNO^+$ $[M+H]^+$ 168.02107, found 168.02116.

*2-Chloro-4-methoxy benzonitrile (**19e**) and 4-chloro-2-methoxy benzonitrile (**19f**)*: **^{1}H NMR** (400 MHz, $CDCl_3$) δ 7.45 (d, *J* = 8.2 Hz, 1H), 6.96 (m, 2H), 3.91 (s, 1H). **^{13}C NMR** (101 MHz, $CDCl_3$) δ 161.8, 140.8, 134.4, 121.4, 115.6, 112.5, 100.5, 56.5 ppm. **HRMS (APCI$^+$)**: For $C_8H_7ClNO^+$ $[M+H]^+$ 168.02107, found 168.02116.

S7 Radical trapping and fluorescence quenching experiments

Radical trapping by TEMPO and APCI-MS detection

Three samples were prepared as follows; 1) a mixture of toluene (140 µmol, 13 mg) and Sc(OTf)$_3$ (5 mol%, 3.4 mg) in 250 µl of CD_3CN, 2) a mixture of toluene (140 µmol, 13 mg) and TEMPO (2 equiv., 280 µmol, 44 mg) in 250 µl of CD_3CN, and 3) a mixture of toluene (140 µmol, 13 mg), Sc(OTf)$_3$ (5 mol%, 3.4 mg) and TEMPO (2 equiv., 280 µmol, 44 mg) in 250 µl of CD_3CN. Subsequently, these mixtures were irradiated by 400 nm LEDs for 24 h. Afterwards, the mixtures were diluted with 400 µl of CD_3CN, and the solutions were injected into APCI-MS in negative (mixture 1) or positive mode (mixtures 1 and 2).

Mixture 1

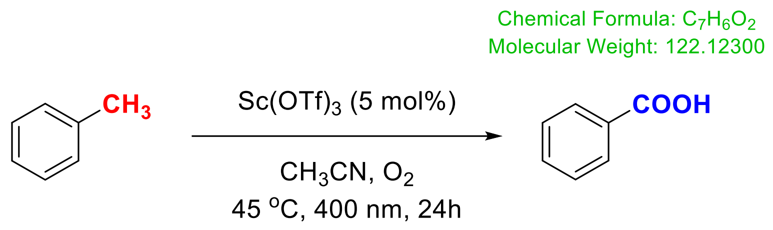

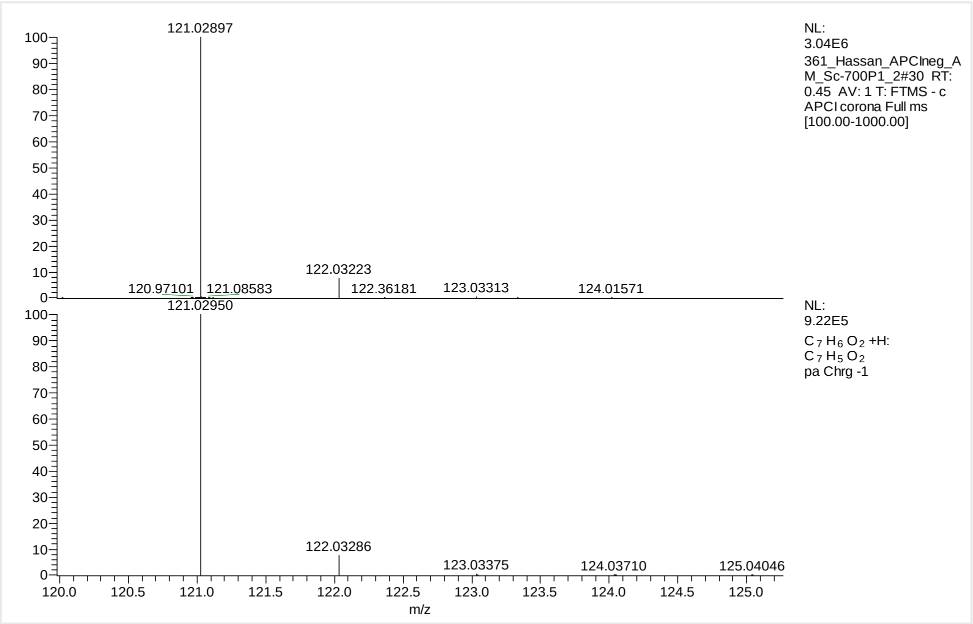

Figure S5. APCI(−)-MS spectrum of a mixture of toluene and Sc(OTf)$_3$ in CD_3CN after irradiation at 400 nm under aerobic conditions.

Mixture 2

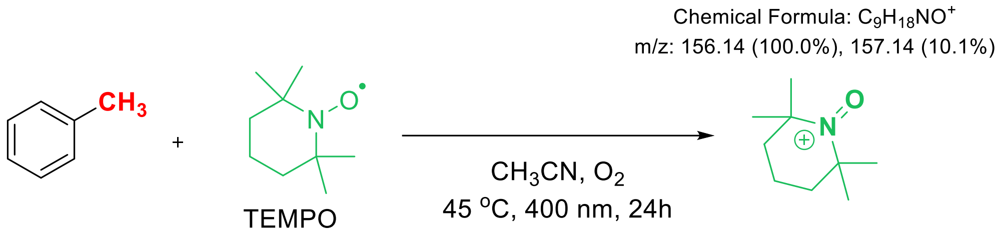

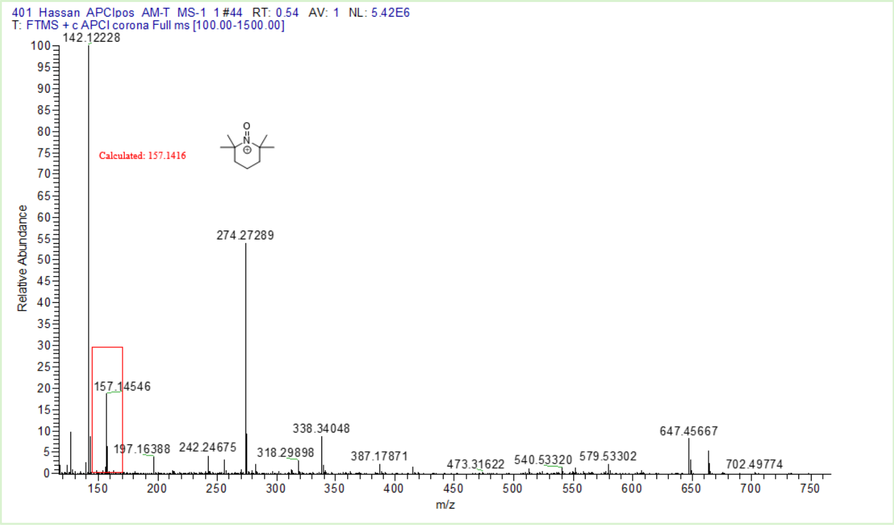

Figure S6. APCI(+)-MS spectrum of a mixture of toluene, TEMPO and $Sc(OTf)_3$ in CD_3CN after irradiation at 400 nm under aerobic conditions.

Mixture 3

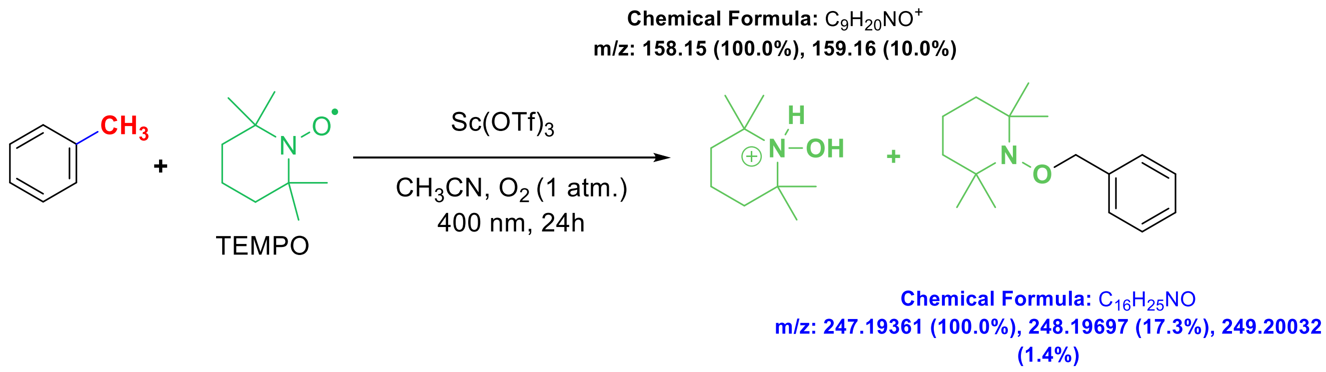

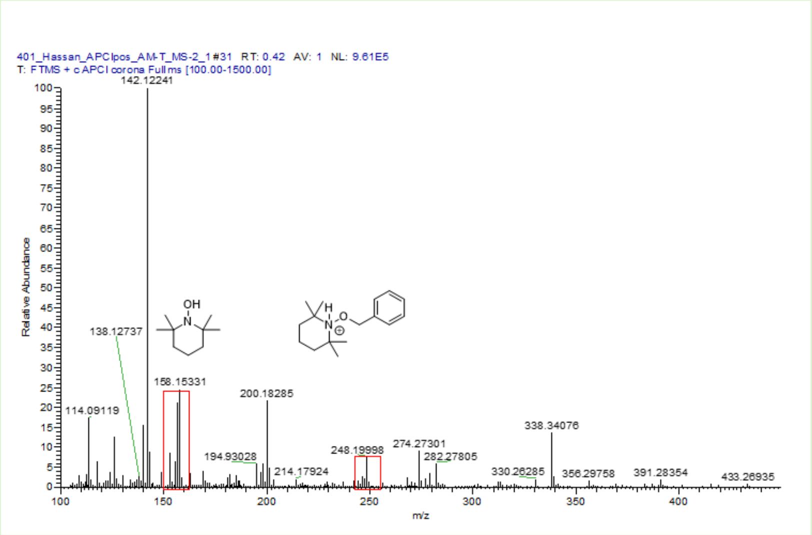

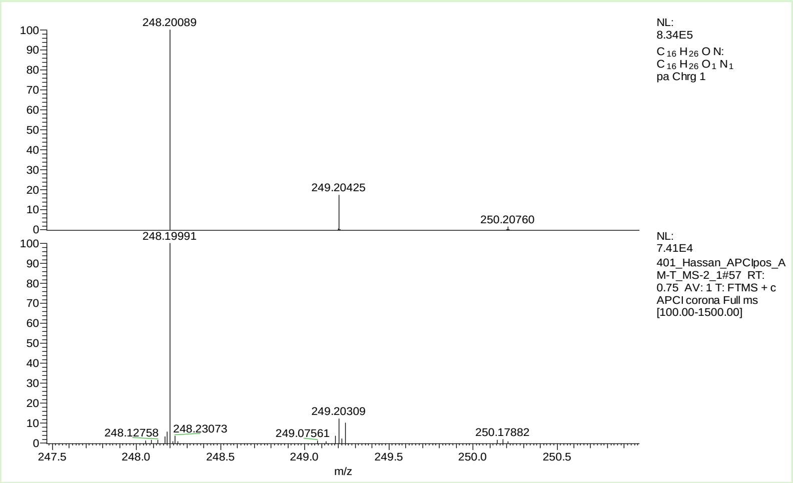

Figure S7. APCI(+)-MS spectrum of a mixture of toluene and TEMPO in CD_3CN after irradiation at 400 nm under aerobic conditions.

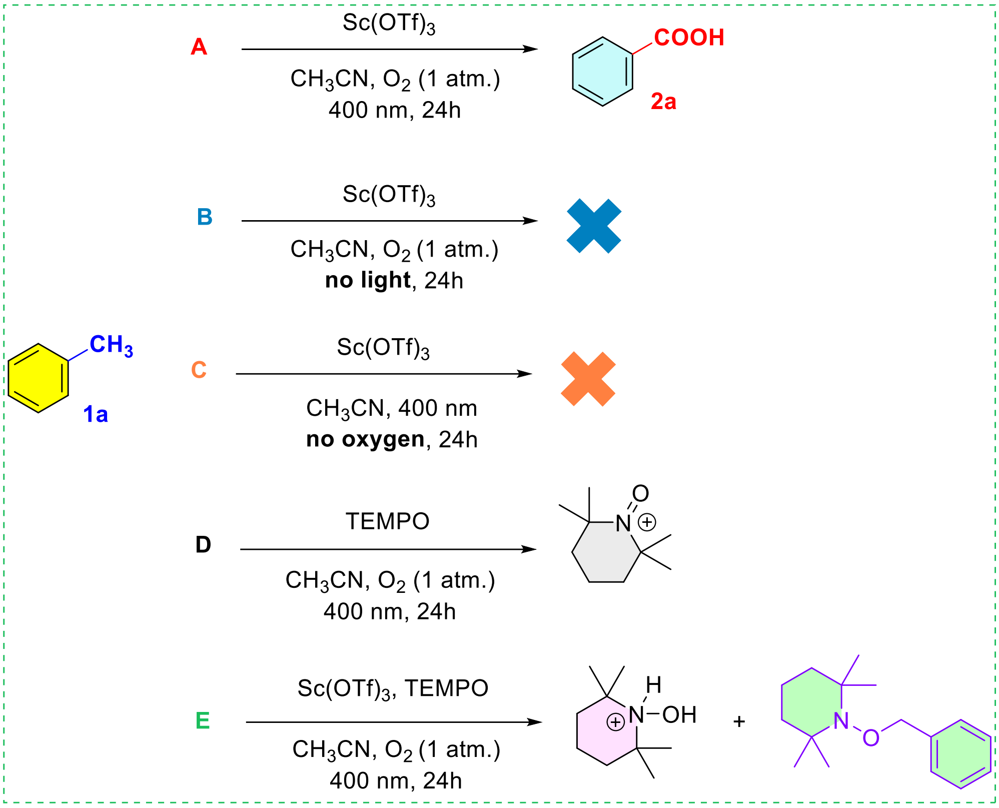

Figure S8. (A) Photooxidation of toluene using $Sc(OTf)_3$, (B) in dark, (C) under anaerobic conditions, (D) radical trapping by TEMPO in the absence of $Sc(OTf)_3$, and (E) radical trapping by TEMPO in the presence of $Sc(OTf)_3$.

Fluorescence quenching measurements (Stern-Volmer experiments)

The relative fluorescence intensities were recorded on Agilent Cary 8454 spectrophotometer. The fluorescence quenching of $Sc(OTf)_3$ (0.02 mol.L^{-1}) by toluene was measured in MeCN at 25 °C. Stern-Volmer plots ($I_o/I=1+K_{SV}[Q]$) were constructed, and the constant K_S was evaluated as the slope of the dependence by using OriginPro 2021 (64-bit) software.

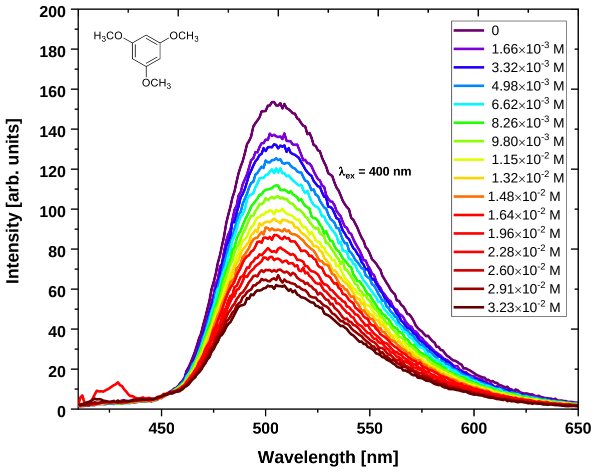

Figure S9. Fluorescence quenching of $[Sc(OTf)_3L_n]^*$ [0.02 mM] with 1,3,5-trimethoxybenzene (**18b**) in MeCN.

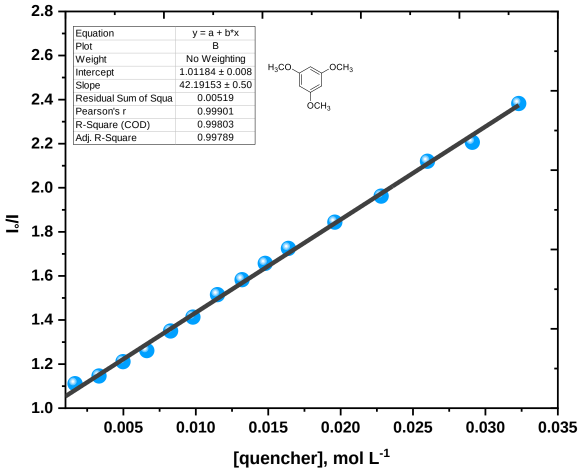

Figure S10. Stern-Volmer plot of fluorescence quenching of $[Sc(OTf)_3L_n]^*$ [0.02 M] with 1,3,5-trimethoxybenzene in MeCN; K_s = 42 L mol^{-1}.

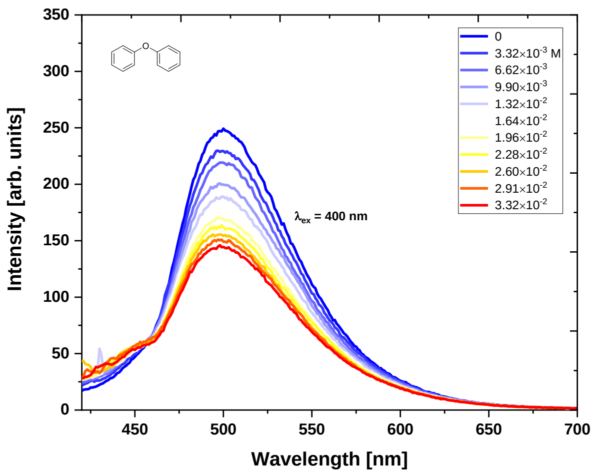

Figure S11. Fluorescence quenching of $[Sc(OTf)_3L_n]^*$ [0.02 M] with diphenyl ether (**18a**) in MeCN.

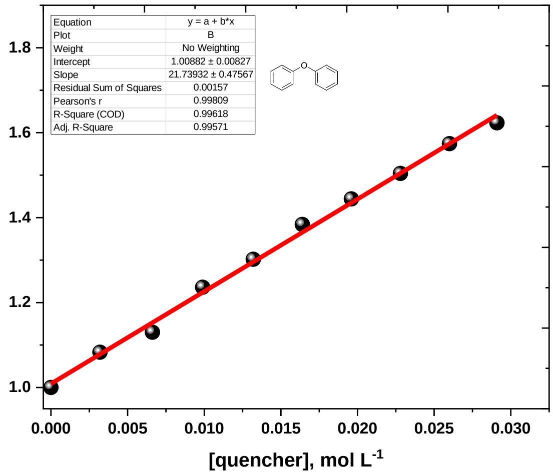

Figure S12. Stern-Volmer plot of fluorescence quenching of $[Sc(OTf)_3L_n]^*$ [0.02 M] with diphenyl ether (**18a**) in MeCN; $K_s = 22$ L mol^{-1}.

S8 Experiment with isotopically labelled oxygen

Discussion: When providing oxidation of toluene (**1a**) with $^{18}O_2$ (percentage of oxygen $^{18}O_2$ > 97%), we obtained benzoic acid in 65 – 87% yield with average isotopic composition 1:30:69 (**2a**/**2a**-^{18}O/**2a**-$^{18}O_2$; see Table S8, Entries 1 and 2). We excluded that atmosphere in our Schlenk tube contains some amount of $^{16}O_2$ which could have entered the mixture during sample preparation. We hypothesised that observed **2a**-^{18}O originated from residual moisture ($H_2{}^{16}O$) which introduces ^{16}O atom into benzaldehyde by hydrate-aldehyde equilibrium. This hypothesis was confirmed by a series of observations: (i) oxygen from water appeared in benzoic acid during toluene oxidation as evident from an experiment with $^{16}O_2$ in the presence of $H_2{}^{18}O$ (Entry 3), (ii) oxygen from water was introduced into benzoic acid during benzaldehyde oxidation as evident from an experiment with $^{16}O_2$ in the presence of $H_2{}^{18}O$ (Entry 5) as well as with $^{18}O_2$ in the presence of $H_2{}^{18}O$ (Entry 6), (iii) some amount (13 %) of benzoic acid **2a** (with no ^{18}O atom) is formed during oxidation of benzaldehyde with $^{18}O_2$.

Finally, oxygen exchange was proven by direct experiment with benzaldehyde and $H_2{}^{18}O$ under standard reaction conditions (acetonitrile, in the presence of scandium triflate) resulting into benzaldehyde/benzaldehyde-^{18}O mixture 73:27 after 24 h stirring under argon atmosphere (Table S9, Entry 1). Importantly, analogous experiment with commercial isotopically labelled benzoic acid (composition determined in independent experiment: **2a**/**2a**-^{18}O/**2a**-$^{18}O_2$ 1:14:85, see Entry 3) did not show any oxygen exchange (Entry 2).

Regarding moisture, we found significantly enhanced (by a factor of 500) water content in the reaction mixture (see Table S10, Entries 2 and 3) in contrast to acetonitrile used in experiments (Entry 1). The main reason for higher water content is probably high oxophilicity of $Sc(OTf)_3$ which tend to bind water either in solid form or in solution.

Experimental: Into Schlenk tube, scandium triflate (3.5 mg, 7 mmol), toluene (16 μL, 140 mmol) and dry acetonitrile (0.25 mL) were added. Schlenk tube was degassed using freeze – thaw – pump technique [cooled in liquid nitrogen, evaporated to 1.1 x 10^{-1} torr, 6 times, filled with isotopic labelled oxygen (97 % $^{18}O_2$), evaporated and repeatedly filled with isotopic labelled oxygen (^{18} O_2)]. Reaction mixture was stirred at 45 °C under irradiation with blue LED (400 nm) for 24 hours. After that, reaction mixture was diluted with 0.5 mL of DMSO-d_6. Conversion was determined using ^{1}H NMR (ratio of integrated aromatic signals of toluene and benzoic acid), isotopic ratio was determined using GC-MS technique [Orbitrap Exploris 240 high resolution mass spectrometer connected to Agilent 7890 gas chromatograph with a DB5ms UI 30m x 0.25 mm, film 0.25 μm capillary column was used for GC-MS analyses. SICRIT (Plasmion GmbH) ionisation was used in positive mode to selectively detect $[M+H]^+$ ions of benzoic acid].

Table S8. Determination of isotopic labelled oxygen in benzoic acid [a]

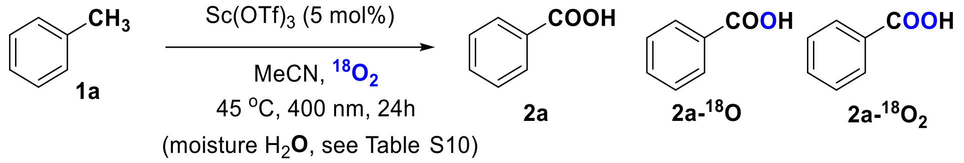

Entry	Yield[b] [%]	Isomers ratio[c] m/z (M+H+) 123 – 125 – 127 [%]
1	87	1 – 33 – 66
2	65	1 – 27 – 72
3	n.d.	46.5 – 52.5 – 1[d]
4	n.d.	13 – 77.5 – 9.5[e]
5	n.d.	51 – 47 – 2[f]
6	n.d.	7 – 44 – 49[g]

[a] Standard reaction conditions: Substrate (0.140 mmol), MeCN (0.25 mL), 45 °C, $^{18}O_2$, blue LED (400 nm), and 24 h. [b] Yields were determined from ^{1}H NMR spectra. [c] Ratio was determined by GC-MS analysis, GC_SICRIT pos or EI pos. [d] Condition: O_2, $H_2{}^{18}O$ (1.5 equiv). [e] Condition: substrate – benzaldehyde (0.140 mmol), $^{18}O_2$. [f] Condition: substrate – benzaldehyde (0.140 mmol), O_2, $H_2{}^{18}O$ (1.5 equiv). [g] Condition: substrate – benzaldehyde (0.140 mmol), $^{18}O_2$, $H_2{}^{18}O$ (1.5 equiv).

Table S9. Investigation of replacement of oxygen in benzaldehyde and benzoic acid.

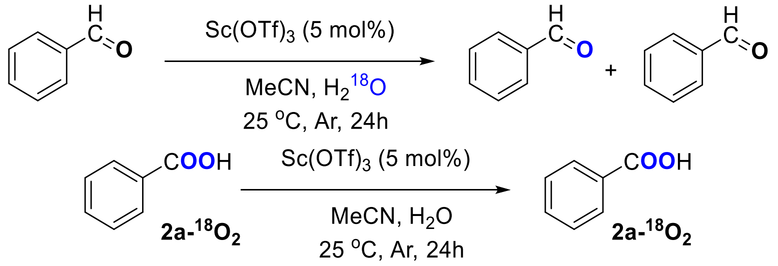

Entry	Isomers ratio[c] m/z (M+H+) 107 – 109 [%]	Isomers ratio[c] m/z (M+H+) 123 – 125 – 127 [%]
1[a]	73 – 27	-
2[b]	-	1 – 14 – 85
3[d]	-	1 – 14 – 85

[a] Standard reaction mixture: benzaldehyde (0.140 mmol), $Sc(OTf)_3$ (0.07 mmol), $H_2{}^{18}O$ (1.5 equiv.), MeCN (0.25 mL). [b] Standard reaction mixture: benzoic acid (0.140 mmol), $Sc(OTf)_3$ (0.07 mmol), $H_2{}^{18}O$ (1.5 equiv.), MeCN (0.25 mL). [c] Ratio was determined by GC-MS analysis, GC_SICRIT pos or EI pos. [d] Benzoic acid -^{18}O (0.140 mmol), MeCN (0.25 mL).

Table S10. Determination of water content by Karl-Fischer method.

Entry	Sample	Sample mg	Water ppm
1	ACN dry	221.65	6.5
2	Reaction mixture[a]	180.58	2241
3	Degassed reaction mixture + $^{18}O_2$	159.8	3398

[a] Standard reaction mixture: toluene (0.140 mmol), $Sc(OTf)_3$ (0.07 mmol), MeCN (0.25 mL).

S9 Cyclic voltammetry measurements

Cyclic voltammetry (CV) was performed in a heart-shaped single-compartment glass cell containing a coiled Ag wire pseudo-reference electrode, a Glassy Carbon microdisc working electrode, and a coiled Pt wire counter electrode. The cell was held inside an earthed Faraday cage and connected to a Metrohm-Autolab PGSTAT302N potentiostat. The internal standard ferrocenium/ferrocene (Fc^+/Fc) was added before the final scans. The sample solutions were 1 mM of analyte dissolved in MeCN containing 10^{-1} M tetrabutylammonium hexafluorophosphate (TBAH). Measurements were performed under an Argon atmosphere and an oxygen atmosphere when bubbled with oxygen 1 min prior to the measurements.

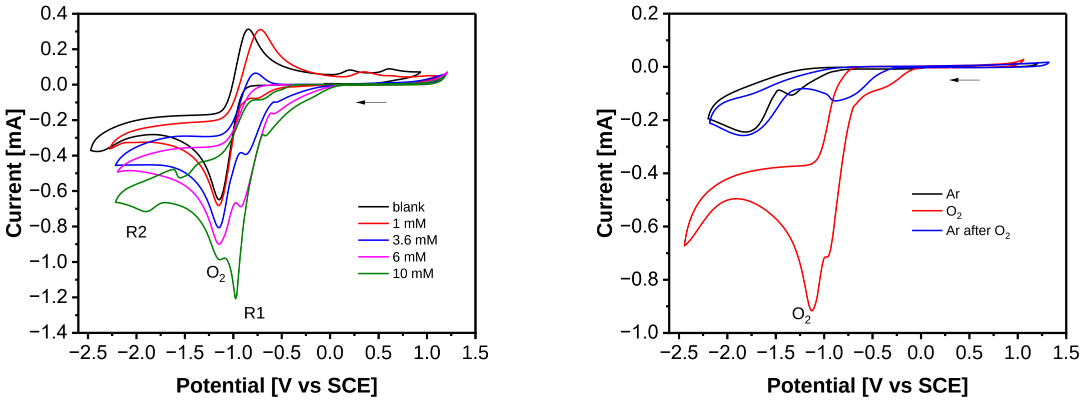

Figure S13. Cyclic voltammograms of oxygen-saturated acetonitrile before (black curve) and after addition of $Sc(OTf)_3$ (left). Cyclic voltammogram of $Sc(OTf)_3$ (6 mM) in acetonitrile under argon (black), after bubbling oxygen (red) and again, after bubbling argon (blue) (right).

Table S11. Cathodic peak potentials[a] (V vs SCE)[b] under argon or oxygen atmospheres

	blank	R1 (Sc-O_2)	O_2	R2 (Sc^{3+}/Sc^{2+})
Ar	–	–	–	–1.83
O_2	–	–0.95	–1.16	–1.895[c]

[a] R1 and R2 are $E_{p,c}$ values. [b] Recalculated values using the factor of 0.382 V (ref. 5). [c] 10 mM concentration

S10 EPR measurements

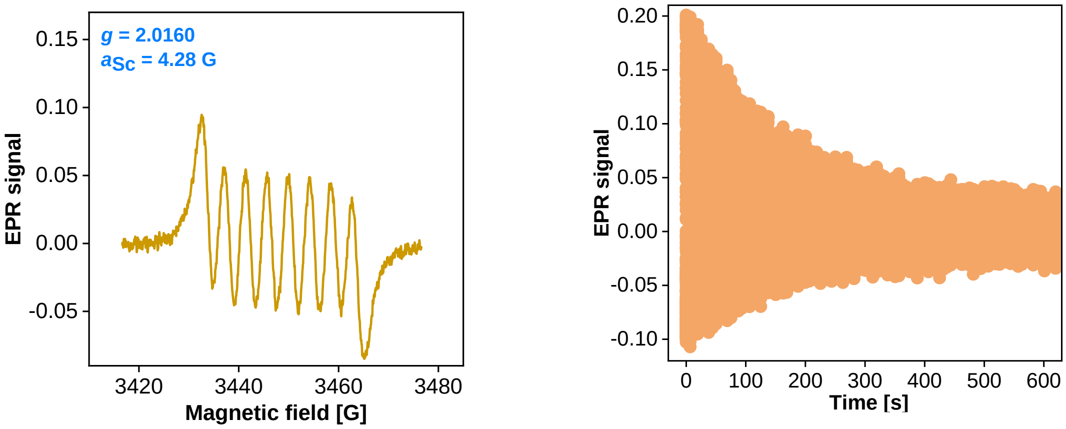

Figure S14. The observed EPR spectra of an oxygen-saturated MeCN solution containing toluene (2 M) and $Sc(OTf)_3$ [0.1 M] upon irradiation with a 400 LED at room temperature (left), and kinetic trace after stopping the irradiation (right).

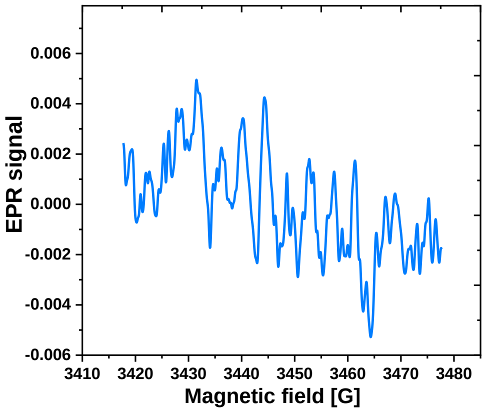

Figure S15. The observed EPR spectrum after 23 minutes of irradiation with a 400 nm LED at room temperature of an oxygen-saturated acetonitrile solution containing, toluene (2 M) and $La(OTf)_3$ (0.1 M).

S11 Determination of hydrogen peroxide in the reaction mixture

The quantification of the reduction product H_2O_2 was assessed using the conventional method of iodide ion titration.[6] After irradiation, a diluted solution of the reaction mixture in MeCN was treated with an excess NaI-acidified MeCN solution and heated at 60 °C for 1 min. The amount of the generated I_3^- was conducted utilising the UV-Vis absorbance spectrum at λ_{max} = 364 nm, recorded on a JASCO V-650 spectrophotometer. The same procedure was carried out on a diluted solution of the reaction mixture before irradiation as a blank. The amount of H_2O_2 formed by the photooxidation of 0.140 mmol of toluene was 0.304 mmol. The excess in the detected H_2O_2 could be attributed to the fact that toluene oxidation into benzoic acid involves several sequential steps.

S12 Quantum chemical calculations – UV-Vis spectra and excited states

All structures were fully optimised to minima, confirmed by frequency calculations having no imaginary frequencies, in Orca software package, release 6.0.1.[7] PBE0 DFT[8] functional was used in the calculation together with Def2-TZVPD basis set. Resolution of identity[7] approach (auxiliary basis sets Def2/J, Def2/JK, Def2-TZVPD/C) was employed to speed up the calculations together with weak long-range interactions.[9] Acetonitrile as a solvent was approximated by CPCM method.[10] Absorption spectra were calculated at the same level of theory using TD-DFT approach (the first 30 excited states were calculated). The optimization of excited S_1, and T_2 states for evaluation of corresponding emissions and higher absorptions was based on TD-DFT or deltaSCF approach (both methods gave almost identical results).[11] Simulated absorption spectra were calculated with line broadening 0.2 eV (see below). For coordinates, see S12. The optimised geometries were used for energy calculation. The energies were calculated at DLPNO-CCSD(T) level of theory[12-15] together with aug-cc-pVTZ basis and corresponding aug-cc-pVTZ/C auxiliary basis set in Orca software package (release 6.0.0). Acetonitrile as a solvent was simulated using CPCM approach.

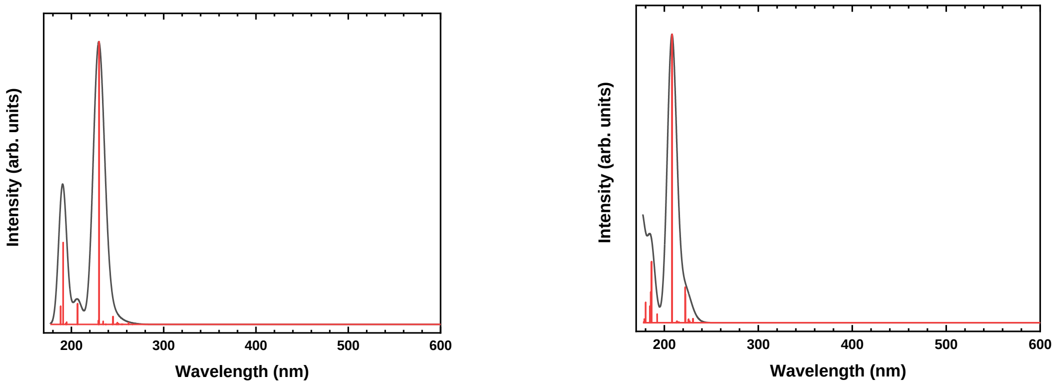

Figure S16. Calculated absorption spectra of hypothetical complexes: $[Sc(MeCN)_2]^{3+}$ S0 (left), $[Sc(MeCN)_3]^{3+}$ S0 (right).

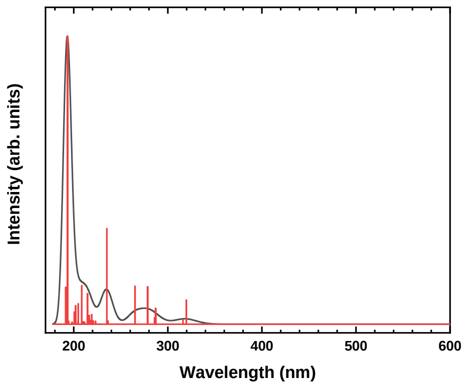

Figure S17. Calculated absorption spectra of hypothetical complexes: $[Sc(MeCN)_2(Tol)]^{3+}$ S0 (left), $[Sc(MeCN)_3(Tol)]^{3+}$ S0 (right).

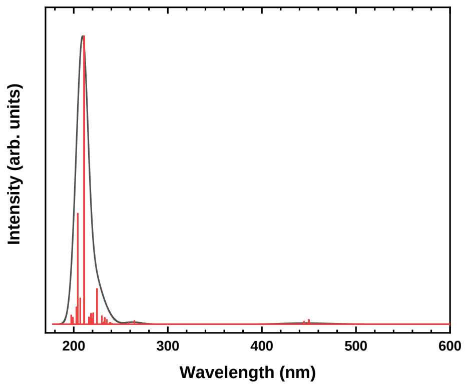

Figure S18. Calculated absorption spectra of hypothetical complexes: $[Sc(MeCN)_2(\eta^2\text{-}O_2)]^{3+}$ S0 (left), $[Sc(MeCN)_3(\eta^2\text{-}O_2)]^{3+}$ S0 (right).

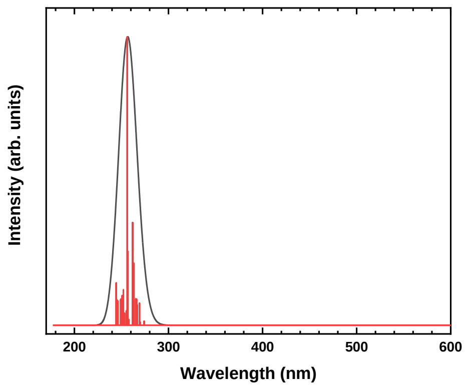

Figure S19. Calculated absorption spectra of hypothetical complexes: $[Sc(MeCN)_2(\eta^1\text{-}O_2)]^{3+}$ T1 (left), $[Sc(MeCN)_3(\eta^1\text{-}O_2)]^{3+}$ T1 (right).

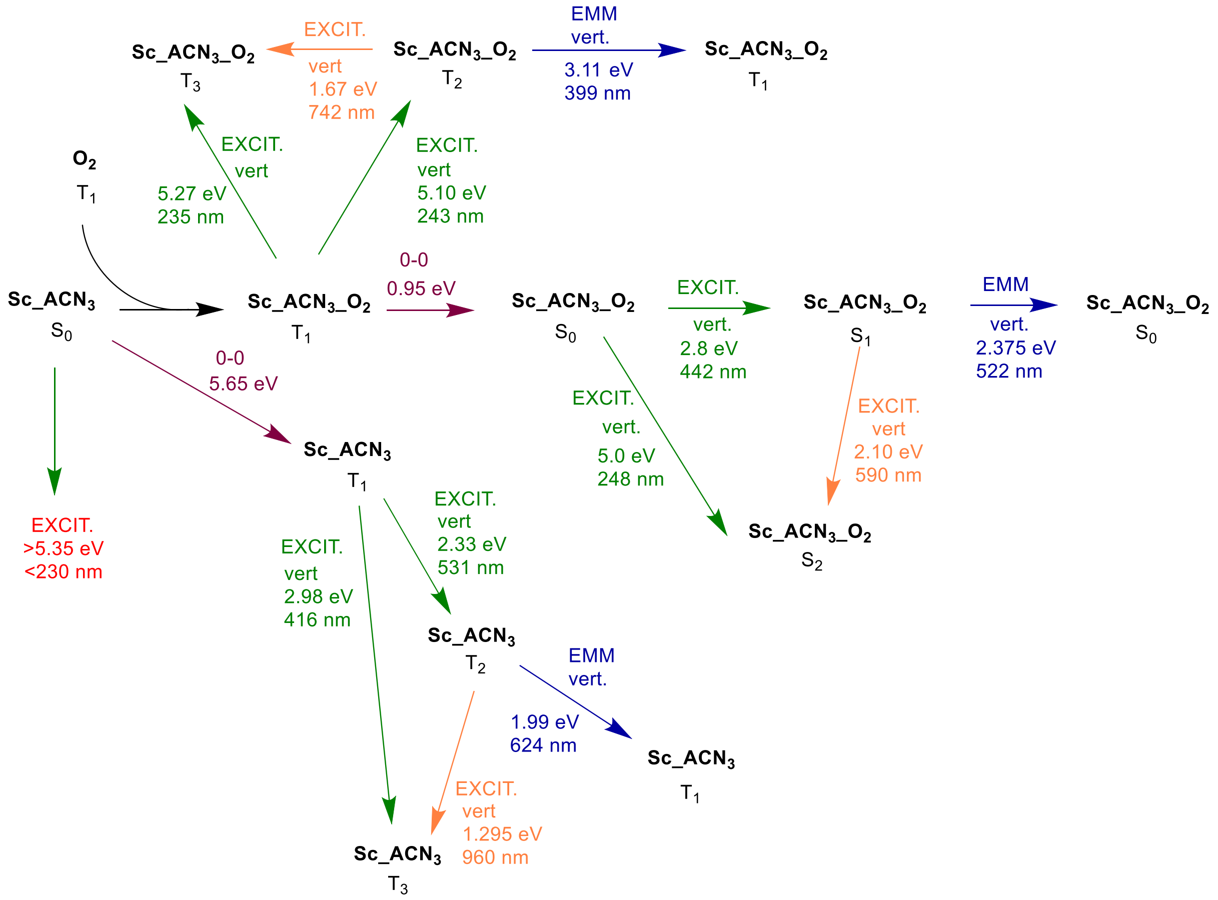

Figure S20. Calculated energies of excited states and corresponding transitions based on TD-DFT or deltaSCF approach at the same level of theory as previously (PBE0/Def2-TZVPD).

S13 NMR spectra of photooxidation and cyanation products

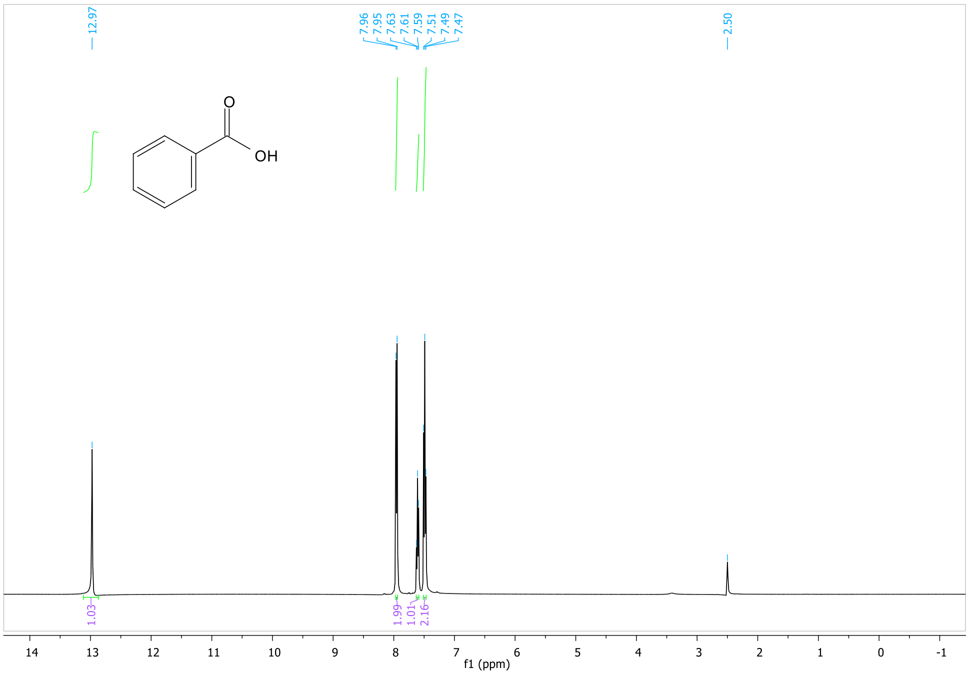

Figure S21. ^{1}H NMR (400 MHz) spectrum of benzoic acid (**2a**) in DMSO-d_6 at 298 K.

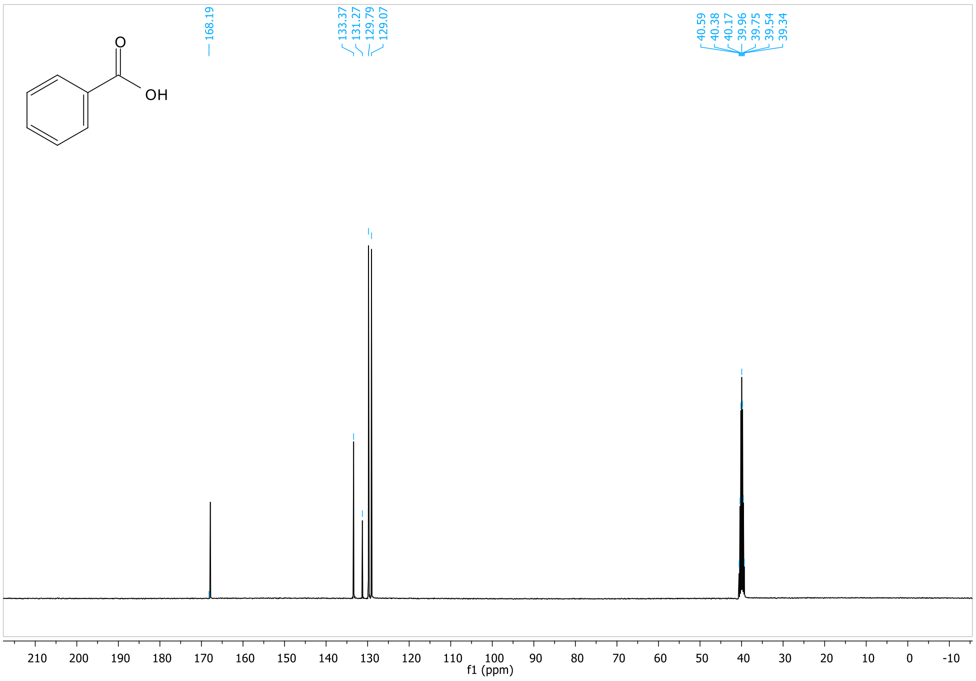

Figure S22. ^{13}C NMR (101 MHz) spectrum of benzoic acid (**2a**) in DMSO-d_6 at 298 K.

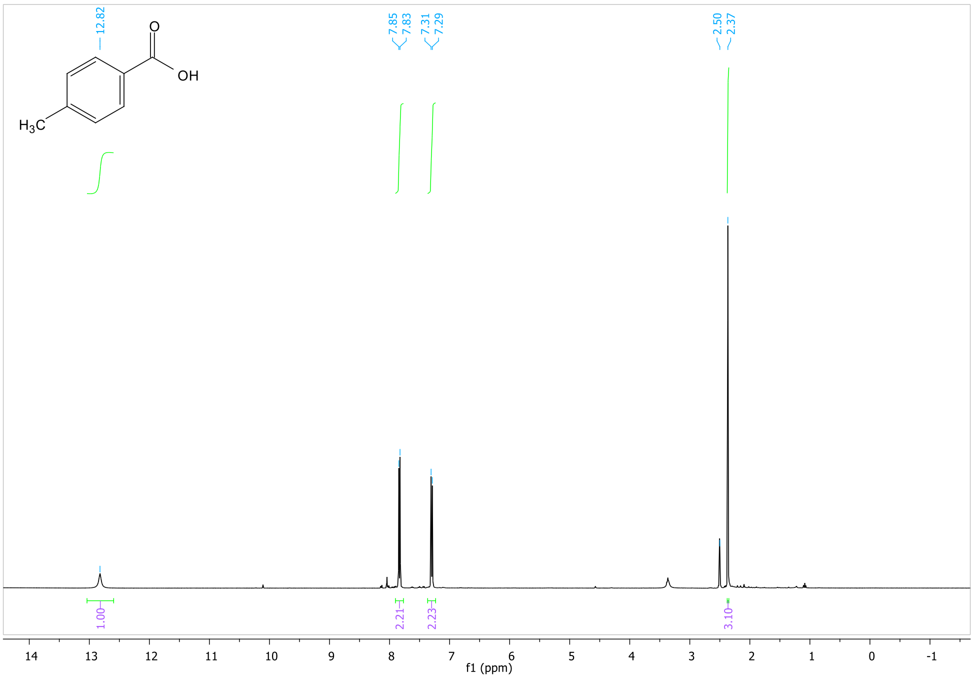

Figure S23. ^{1}H NMR (400 MHz) spectrum of 4-methyl benzoic acid (**2f**) in DMSO-d$_6$ at 298 K.

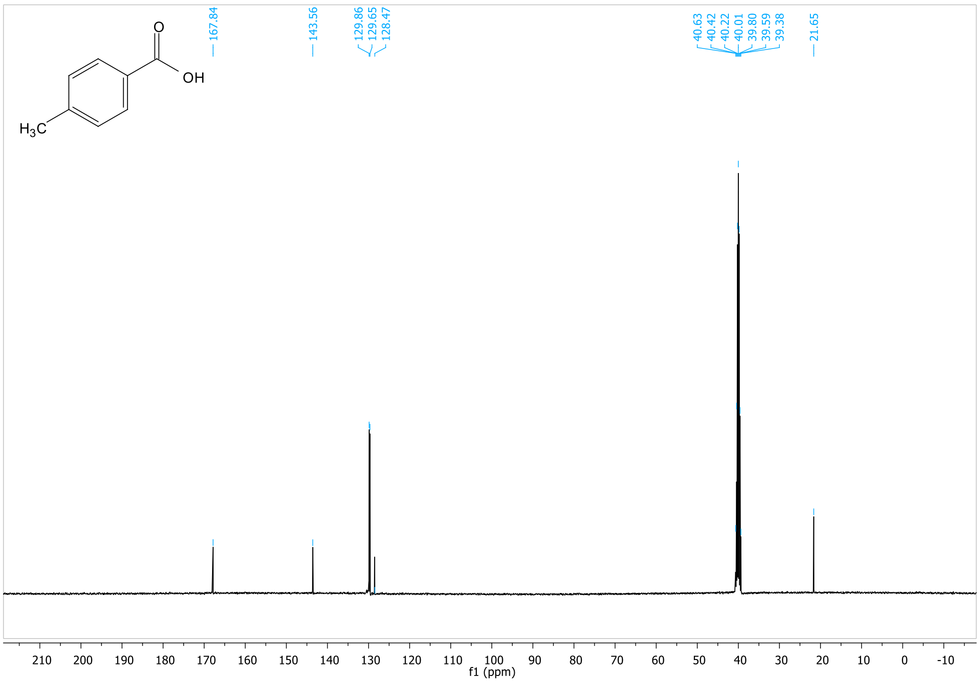

Figure S24. ^{13}C NMR (101 MHz) spectrum of 4-methyl benzoic acid (**2f**) in DMSO-d$_6$ at 298 K.

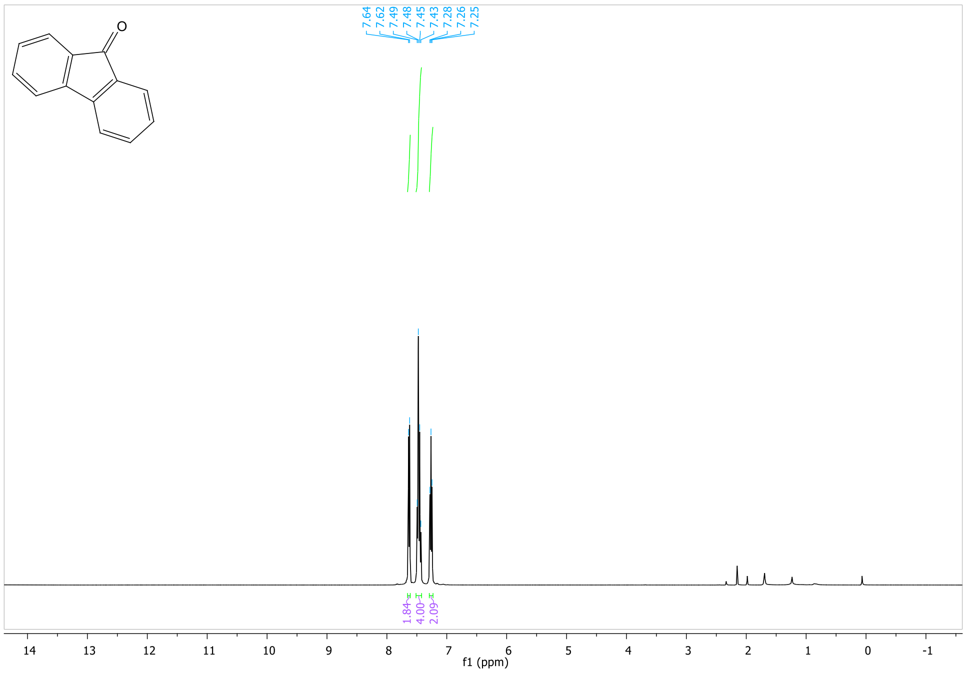

Figure S25. ^{1}H NMR (400 MHz) spectrum of 9-fluorenone (**9**) in $CDCl_3$ at 298 K.

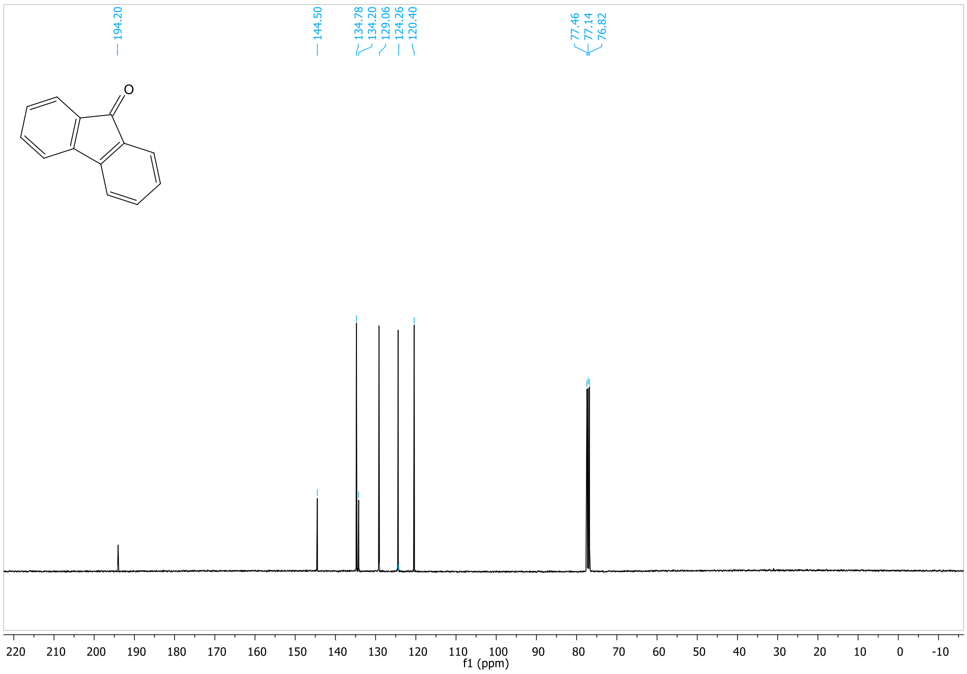

Figure S26. ^{13}C NMR (101 MHz) spectrum of 9-fluorenone (**9**) in $CDCl_3$ at 298 K.

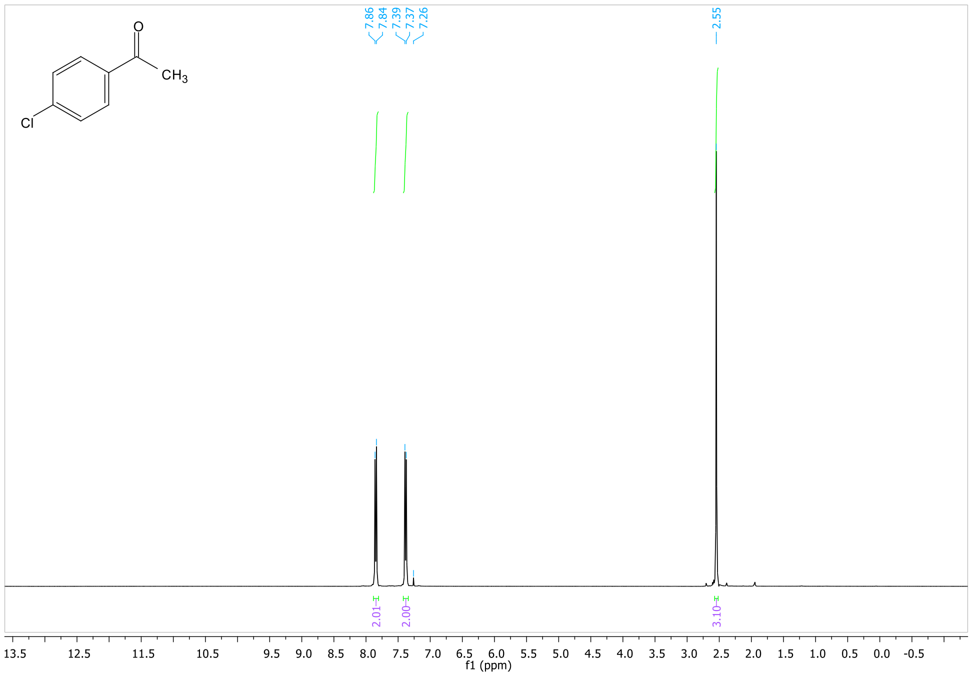

Figure S27. ^{1}H NMR (400 MHz) spectrum of 4-chloroacetophenone (**13b**) in $CDCl_3$ at 298 K.

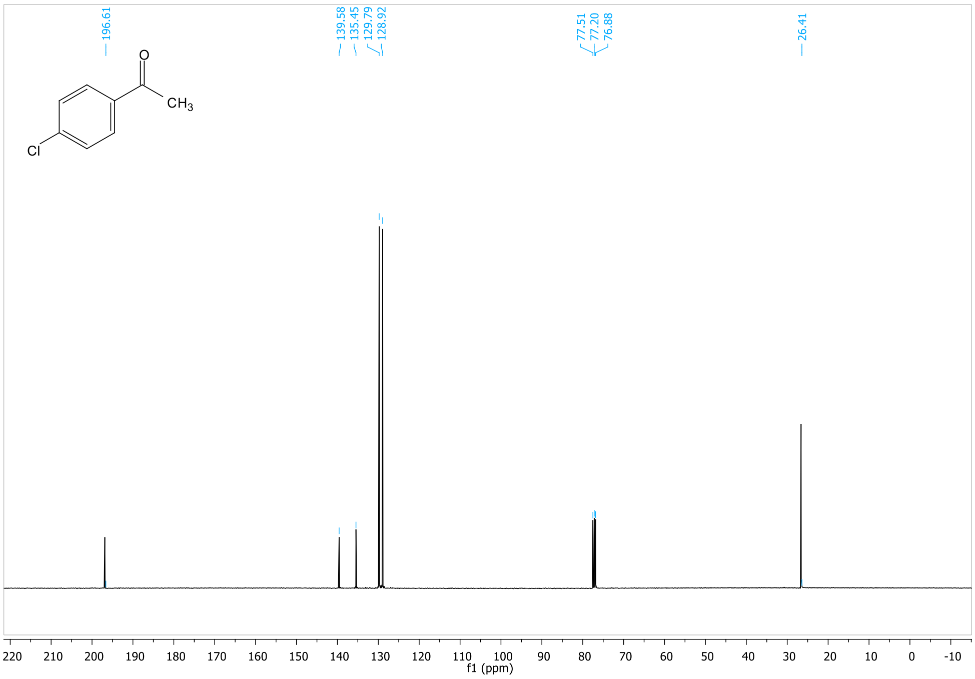

Figure S28. ^{13}C NMR (101 MHz) spectrum of 4-chloroacetophenone (**13b**) in $CDCl_3$ at 298 K.

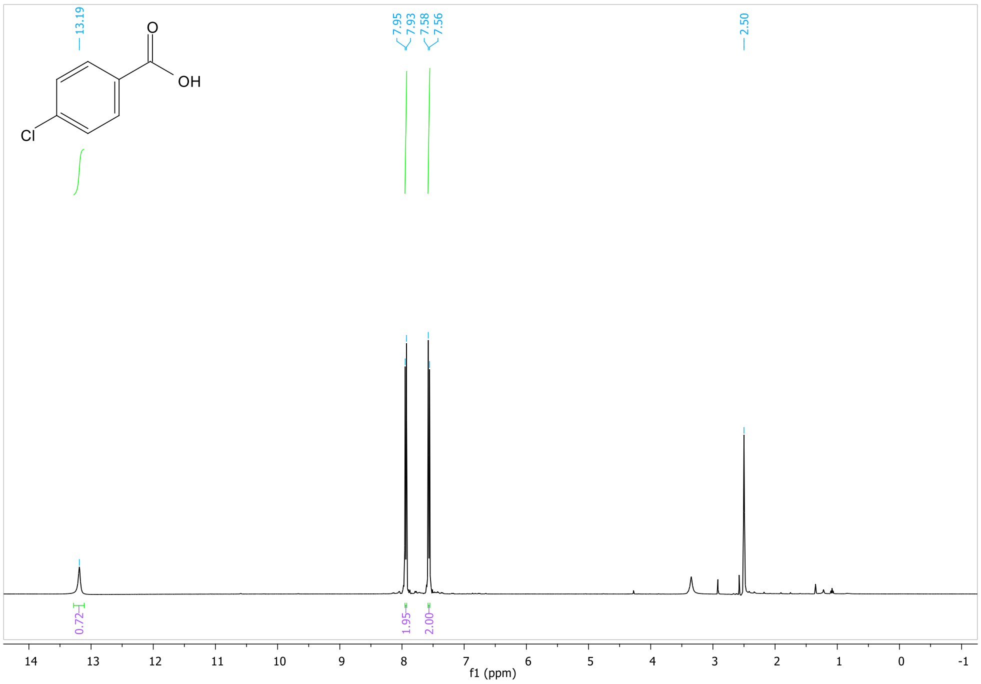

Figure S29. ^{1}H NMR (400 MHz) spectrum of 4-chlorobenzoic acid (**2b**) in DMSO-d_6 at 298 K.

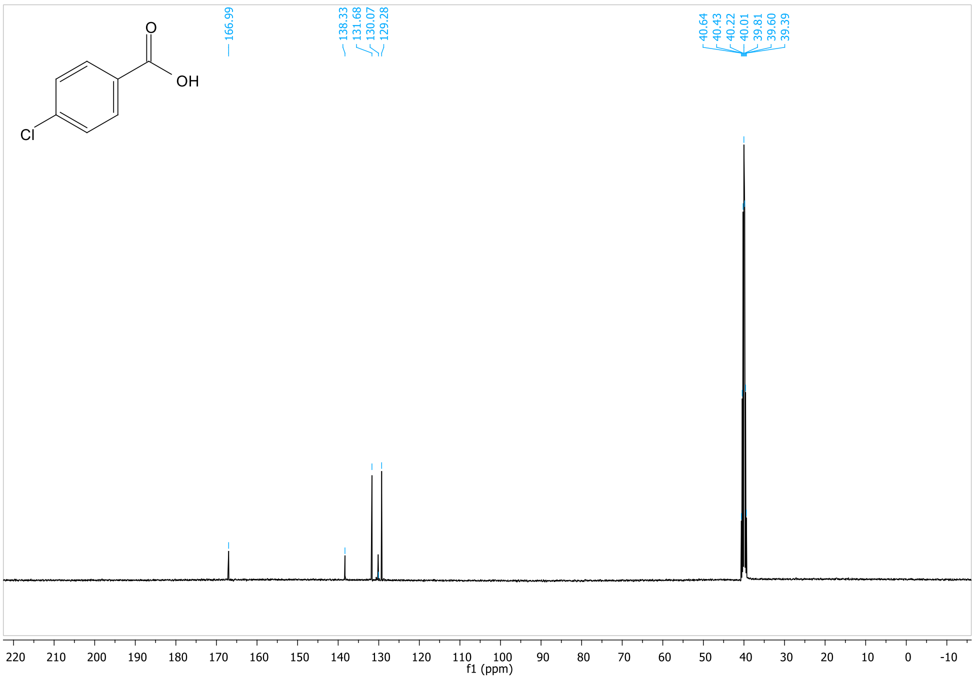

Figure S30. ^{13}C NMR (101 MHz) spectrum of 4-chlorobenzoic acid (**2b**) in DMSO-d_6 at 298 K.

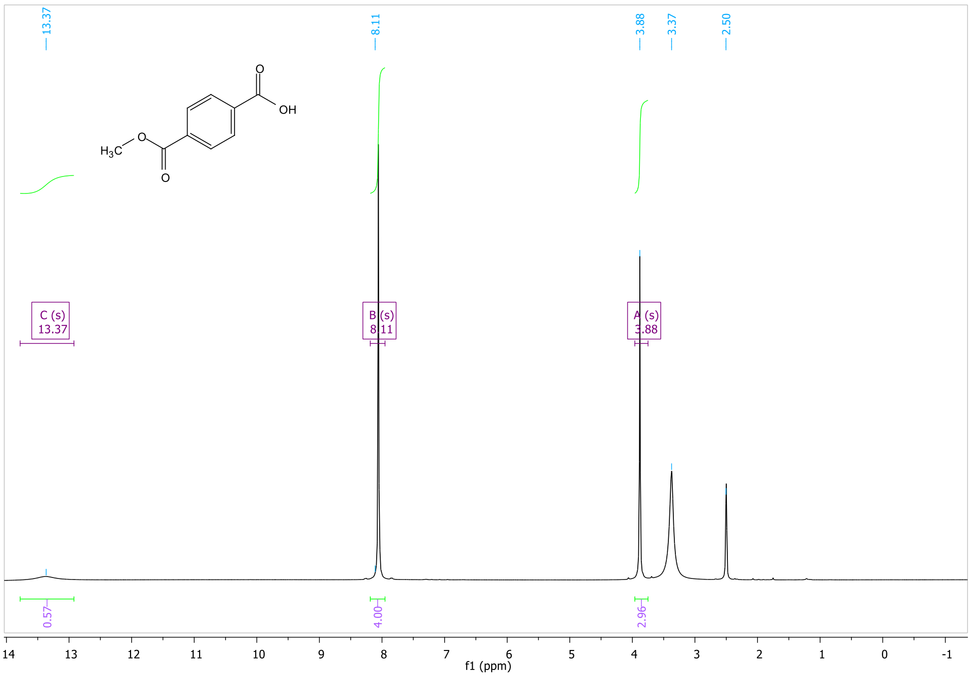

Figure S31. ^{1}H NMR (400 MHz) spectrum of 4-(methoxycarbonyl)benzoic acid (**2d**) in DMSO-d$_6$ at 298 K.

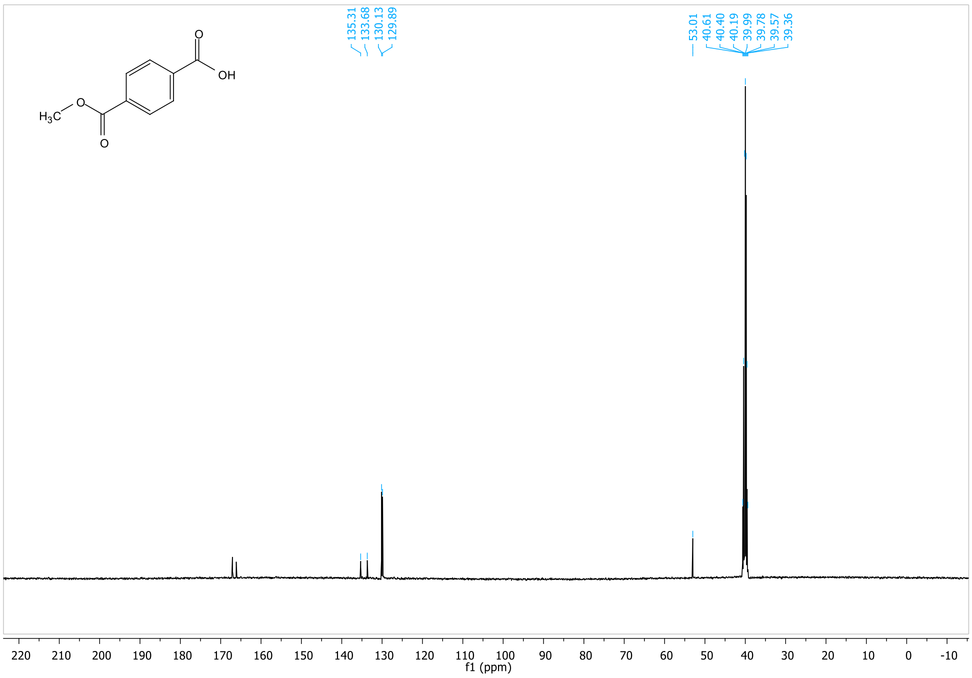

Figure S32. ^{13}C NMR (101 MHz) spectrum of 4-(methoxycarbonyl)benzoic acid (**2d**) in DMSO-d$_6$ at 298 K.

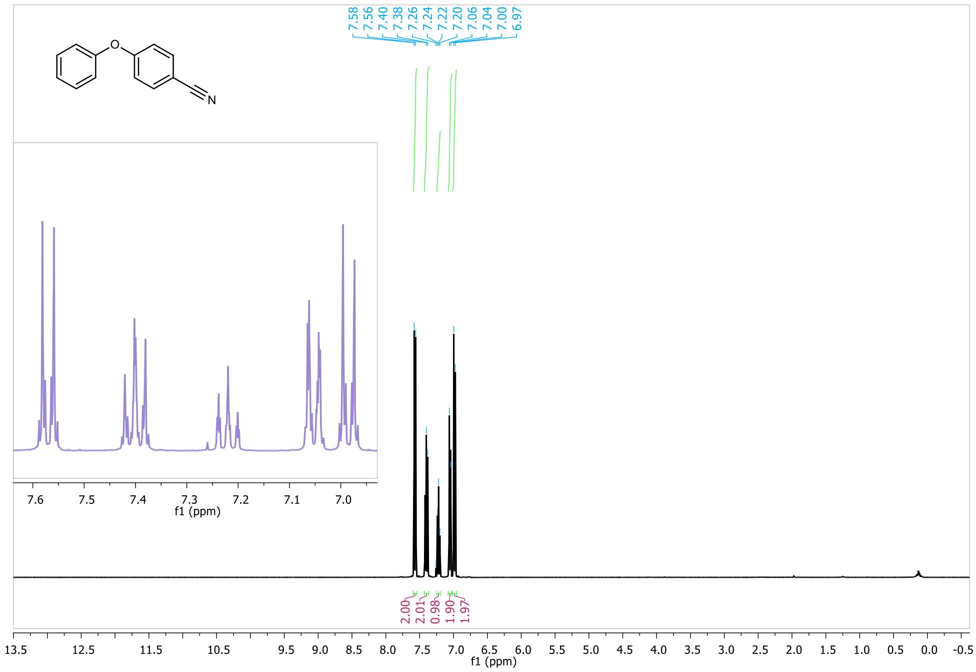

Figure S33. ^{1}H NMR (400 MHz) spectrum of 4-phenoxybenzonitrile (**19a**) in $CDCl_3$ at 298 K.

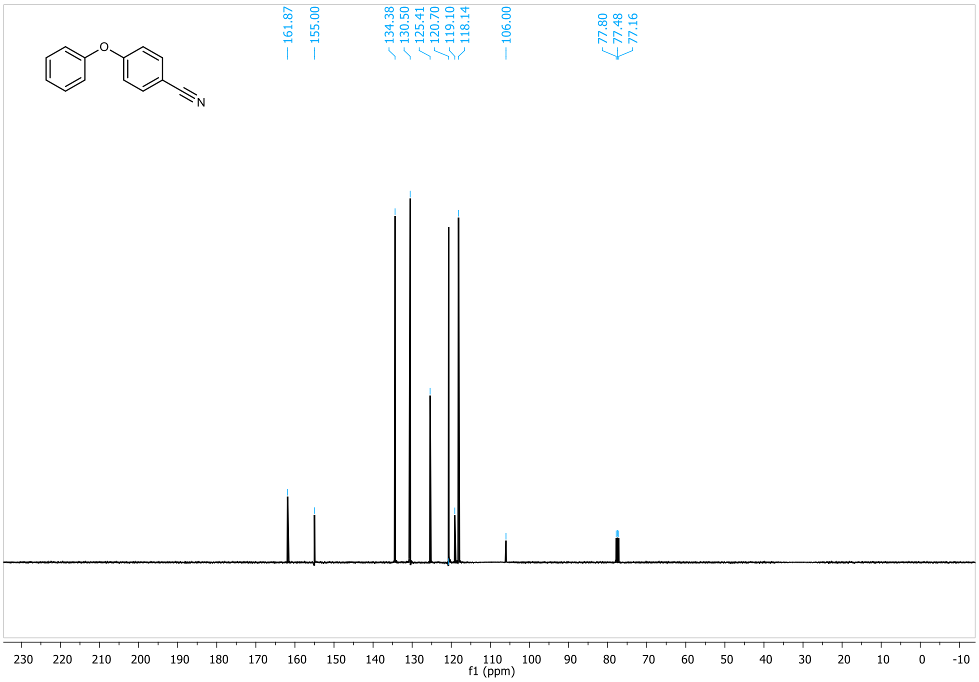

Figure S34. ^{13}C NMR (101 MHz) of 4-phenoxybenzonitrile (**19a**) in $CDCl_3$ at 298 K.

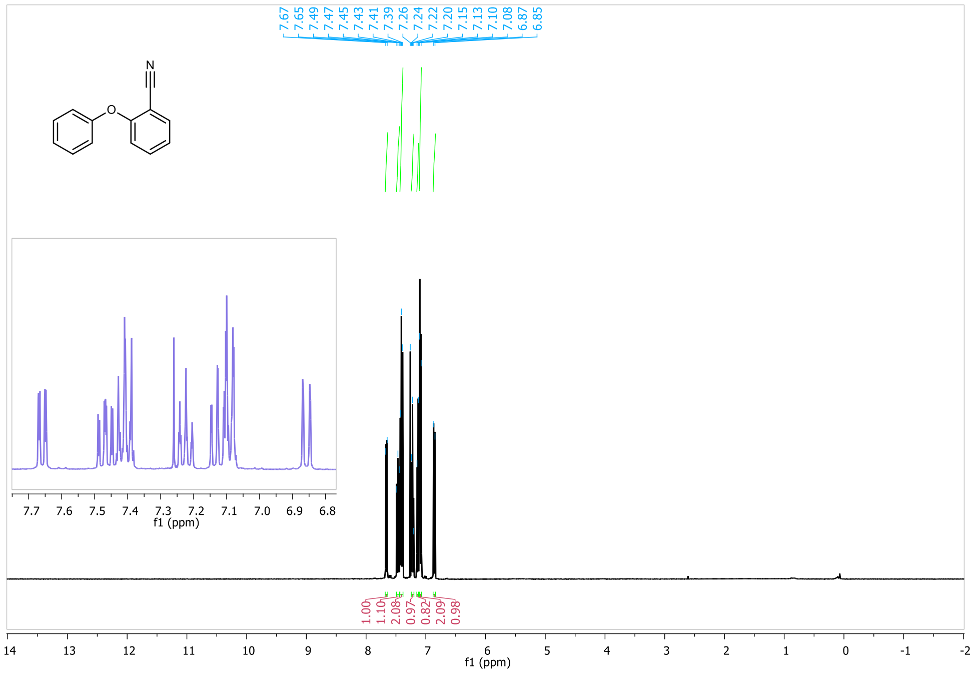

Figure S35. ^{1}H NMR (400 MHz) spectrum of 2-phenoxybenzonitrile (**19b**) in $CDCl_3$ at 298 K.

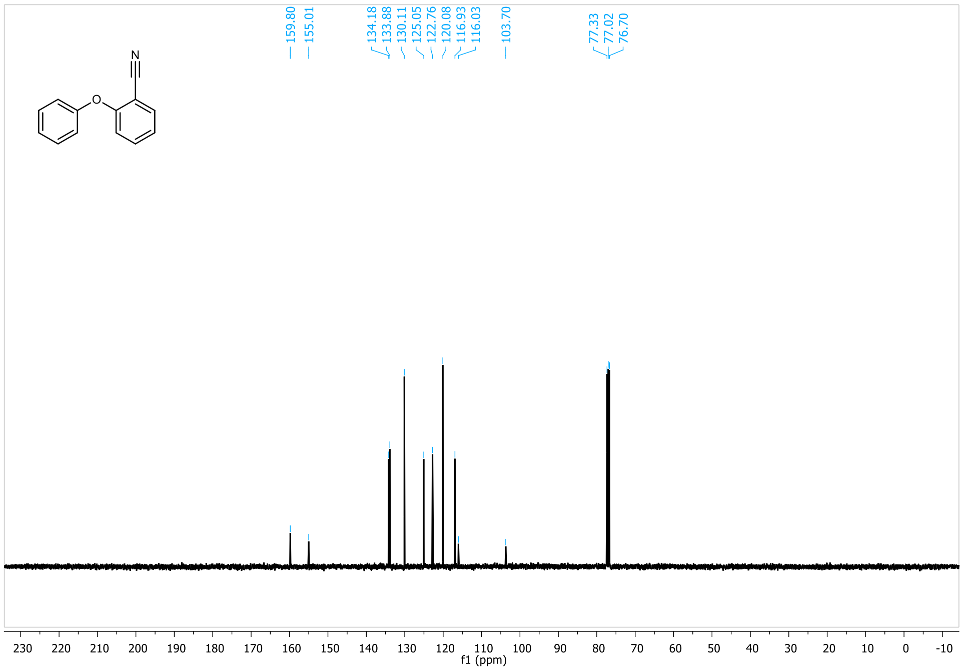

Figure S36. ^{13}C NMR (101 MHz) spectrum of 2-phenoxybenzonitrile (**19b**) in $CDCl_3$ at 298 K.

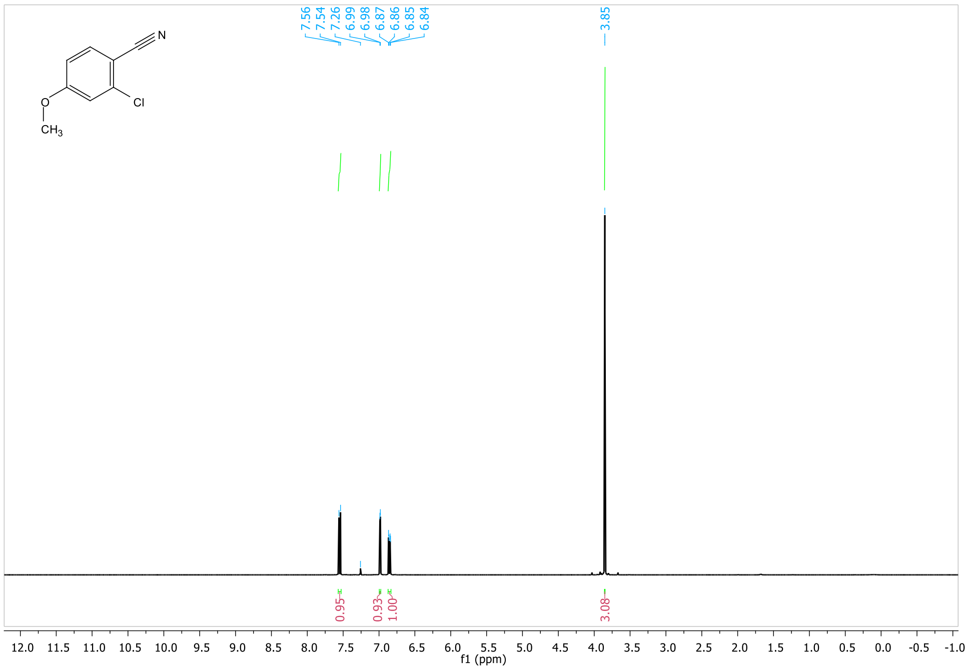

Figure S37. ^{1}H NMR (400 MHz) spectrum of 2-chloro-4-methoxy benzonitrile (**19e**) in $CDCl_3$ at 298 K.

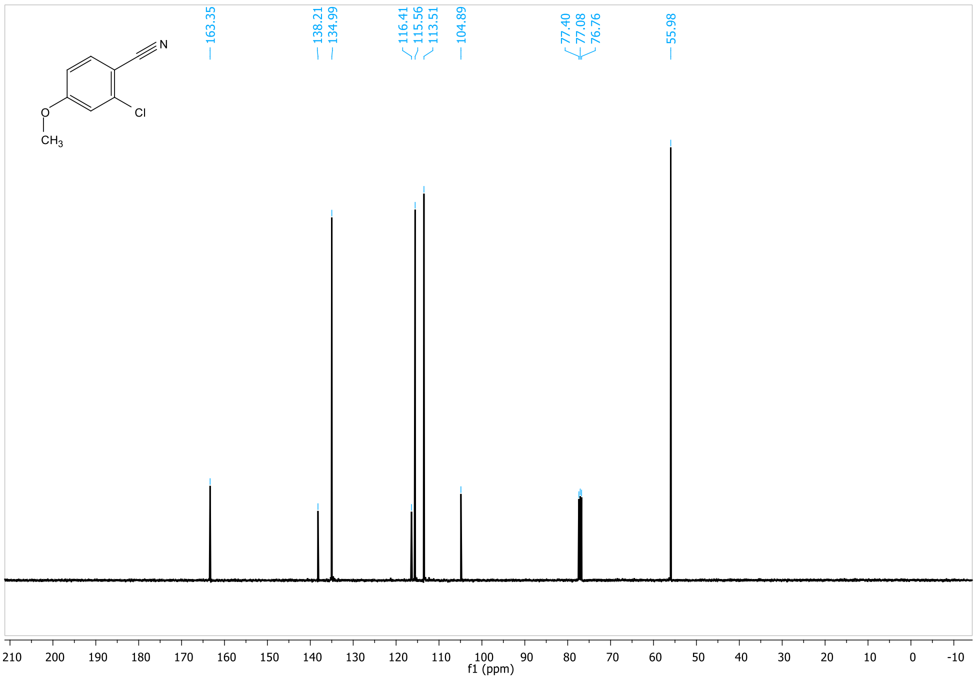

Figure S38. ^{13}C NMR (101 MHz) spectrum of 2-chloro-4-methoxy benzonitrile (**19e**) in $CDCl_3$ at 298 K.

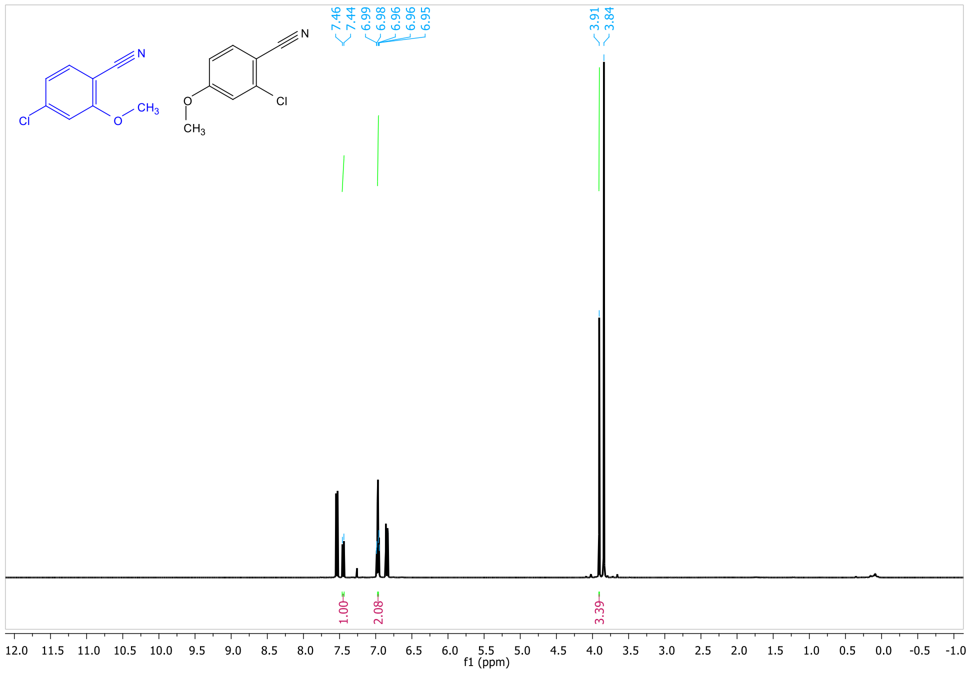

Figure S39. ^{1}H NMR (400 MHz) spectrum of a mixture of 2-chloro-4-methoxy benzonitrile (**19f**) and 4-chloro-2-methoxy benzonitrile (**13g**) in $CDCl_3$ at 298 K.

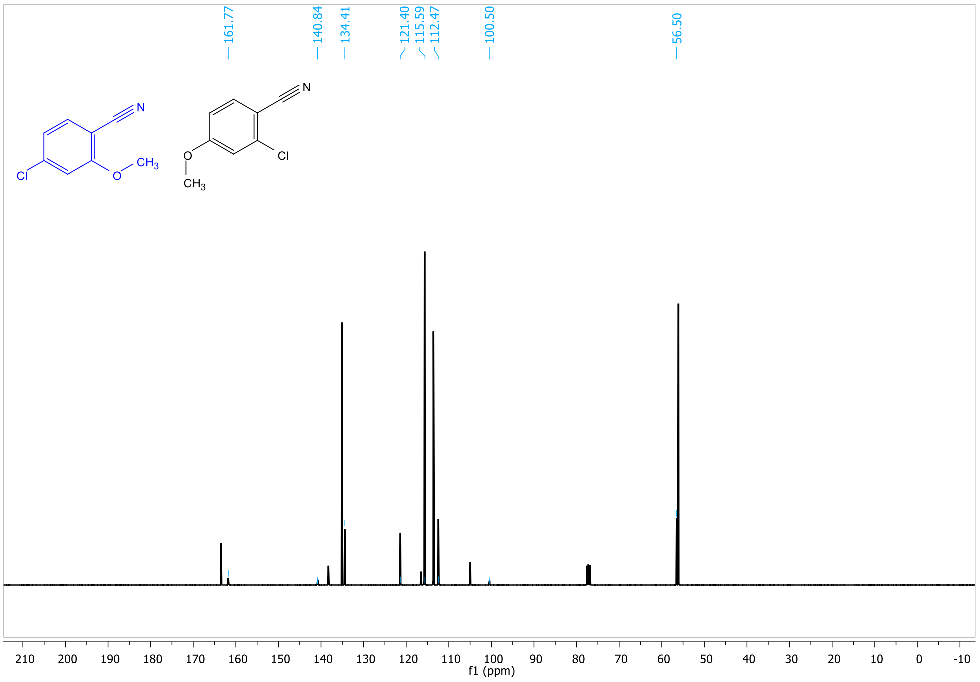

Figure S40. ^{13}C NMR (101 MHz) spectrum of a mixture of 2-chloro-4-methoxy benzonitrile (**19f**) and 4-chloro-2-methoxy benzonitrile (**13g**) in $CDCl_3$ at 298 K.

S14 Experimental setup of photocatalytic experiments

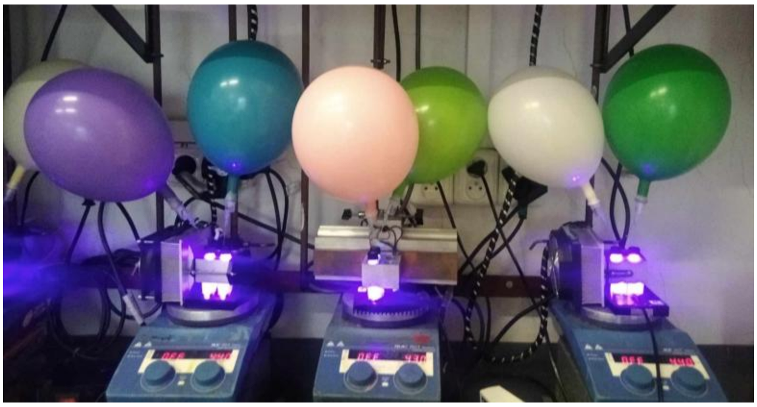

Figure S41. Photochemical setup for reactions on analytical scale.

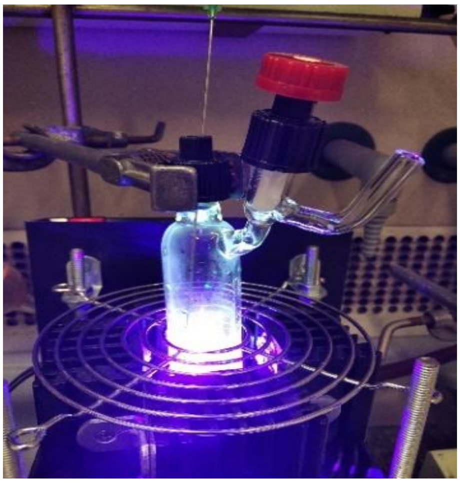

Figure S42. Photochemical setup for reactions on preparative scale (balloon was attached to Schlenk tube).

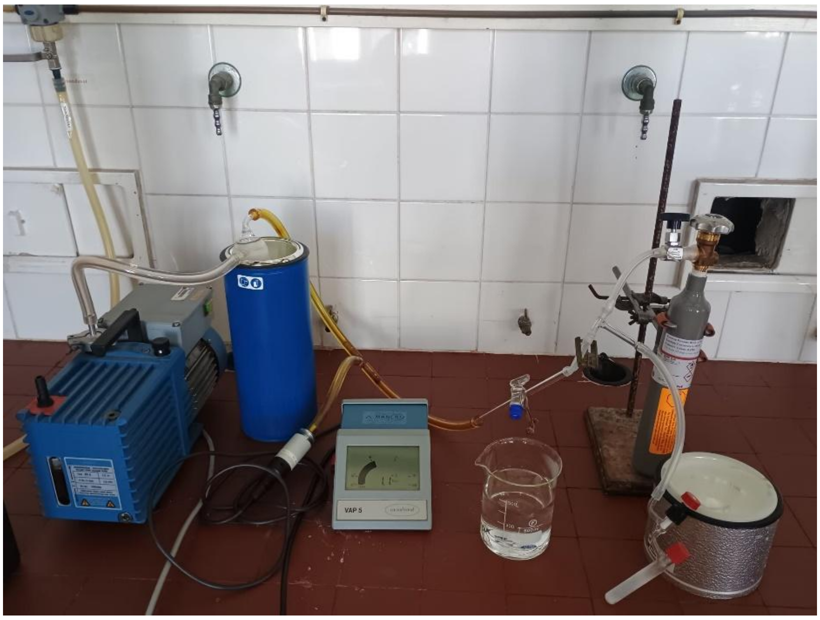

Figure S43. Apparatus for freeze – thaw – pump technique.

References

1 Mart, A. & Shashidhar, M. S. Elaboration of the ether cleaving ability and selectivity of the classical Pearlman's catalyst [Pd (OH) 2/C]: concise synthesis of a precursor for a myo-inositol pyrophosphate. *Tetrahedron* **68**, 9769-9776 (2012).

2 Yuan, H., Sun, M., Zhang, T., Wu, G. & Zhang, Y. Synthesis of Aryl Carboxylic Acids through Ambient Electro-oxidation of Arylacetylenes. *Synthesis* **56**, 179-186 (2023).

3 Hurst, T. E. *et al.* Sodium methyl carbonate as an effective C1 synthon. Synthesis of carboxylic acids, benzophenones, and unsymmetrical ketones. *Org. Lett.* **21**, 3882-3885 (2019).

4 Marcé, P., Lynch, J., Blacker, A. J. & Williams, J. M. Conversion of nitroalkanes into carboxylic acids via iodide catalysis in water. *Chem. Commun.* **52**, 1013-1016 (2016).

5 Aranzaes,J. R., Daniel, M.-C., Astruc D., Metallocenes as references for the determination of redox potentials by cyclic voltammetry - Permethylated iron and cobalt sandwich complexes, inhibition by polyamine dendrimers, and the role of hydroxy-containing ferrocenes. *Can. J. Chem.*, **84**, 288-299 (2006).

6 Fukuzumi, S. *et al.* Efficient Catalysis of Rare-Earth Metal Ions in Photoinduced Electron-Transfer Oxidation of Benzyl Alcohols by a Flavin Analogue. *J. Phys. Chem. A* **105**, 10501-10510 (2001).

7 Neese, F. Software update: The ORCA program system—Version 5.0. *WIREs Computational Molecular Science* **12**, e1606 (2022).

8 Adamo, C. & Barone, V. Toward reliable density functional methods without adjustable parameters: The PBE0 model. *J. Chem. Phys.* **110**, 6158-6170 (1999).

9 Grimme, S., Ehrlich, S. & Goerigk, L. Effect of the damping function in dispersion corrected density functional theory. *J. Comput. Chem.* **32**, 1456-1465 (2011).

10 Garcia-Ratés, M. & Neese, F. Efficient implementation of the analytical second derivatives of hartree–fock and hybrid DFT energies within the framework of the conductor-like polarizable continuum model. *J. Comput. Chem.* **40**, 1816-1828 (2019).

11 Selenius, E., Sigurdarson, A. E., Schmerwitz, Y. L. A., Levi, G. Orbital-Optimized Versus Time-Dependent Density Functional Calculations of Intramolecular Charge Transfer Excited States *J. Chem. Theory Comput.*, **20**, 3809-3822 (2024).

12 Garcia-Ratés, M., Becker, U. & Neese, F. Implicit solvation in domain based pair natural orbital coupled cluster (DLPNO-CCSD) theory. *J. Comput. Chem.* **42**, 1959-1973 (2021).

13 Bistoni, G. *et al.* Treating Subvalence Correlation Effects in Domain Based Pair Natural Orbital Coupled Cluster Calculations: An Out-of-the-Box Approach. *J. Chem. Theor. Comp.* **13**, 3220-3227 (2017).

14 Saitow, M., Becker, U., Riplinger, C., Valeev, E. F. & Neese, F. A new near-linear scaling, efficient and accurate, open-shell domain-based local pair natural orbital coupled cluster singles and doubles theory. *J. Chem. Phys.* **146** (2017).

15 Neese, F., Wennmohs, F. & Hansen, A. Efficient and accurate local approximations to coupled-electron pair approaches: An attempt to revive the pair natural orbital method. *J. Chem. Phys.* **130** (2009).
