# Supplementary material for: Redox-innocent scandium(III) as the sole catalyst in visible light photooxidations
Source: Nat Commun. 2025 Aug 22;16:7851. doi: 10.1038/s41467-025-63233-4 (PMC12373787; doi:10.1038/s41467-025-63233-4)
Supplement: Supplementary file 2 — Description of Additional Supplementary Files [file 41467_2025_63233_MOESM2_ESM.pdf]

File Name: Supplementary Data 1

Description: Geometrics of calculated molecules
